# Song and dance: a memetic angle on the evolution of musicality and music via case studies of a musemeplex in Saint-Saëns and ABBA

**DOI:** 10.3389/fpsyg.2023.1260262

**Published:** 2024-02-28

**Authors:** Steven Jan

**Affiliations:** Department of Music and Design Arts, University of Huddersfield, Huddersfield, United Kingdom

**Keywords:** memetics, museme, musemeplex, ABBA, Saint-Saëns, Implication-Realisation (I-R) theory, image schemata, embodiment

## Abstract

Applying the theory of memetics to music offers the prospect of reconciling general Darwinian principles with the style and structure of music. The nature of the units of cultural evolution in music—memes or, more specifically, musemes—can potentially shed light on the evolutionary processes and pressures attendant upon early-hominin musicality. That is, primarily conjunct, narrow-tessitura musemes (those conforming to Ratner's “singing style,” and its instrumental assimilations) and primarily disjunct, wide-tessitura musemes (those conforming to Ratner's “brilliant style,” and its vocal assimilations) appear to be the outcome of distinct cultural-evolutionary processes. Moreover, musemes in each category arguably acquire their fecundity (perceptual-cognitive salience, and thus transmissibility) by appealing to different music-underpinning brain and body subsystems. Given music's status as an embodied phenomenon, both singing-style and brilliant-style musemes recruit and evoke image schemata, but those in the former category draw primarily upon vocal images of line, direction and continuity; whereas those in the latter category draw primarily upon rhythmic impetus and energy. These two museme-categories may have been molded by distinct biological-evolutionary processes—the evolution of fine vocal control, and that of rhythmic synchronisation, respectively; and they might—via the process of memetic drive—have themselves acted as separate and distinct selection pressures on biological evolution, in order to optimize the environment for their replication. As a case-study of (primarily) singing-style musemes, this article argues that a passage from the love duet “Mon cœur s'ouvre à ta voix” from Camille Saint-Saëns' opera *Samson et Dalila* op. 47 (1877) is the cultural-evolutionary antecedent of the Introduction/Chorus/Outro material of ABBA's song “The Winner Takes It All.” Discussion of their melodic and harmonic similarities supports a memetic link between elements of Saint-Saëns' duet and ABBA's song. These relationships of cultural transmission are argued to have been impelled by the fecundity of the shared musemes, which arises from the image-schematic and embodied effects of the implication-realisation structures (in Narmour's sense) that comprise them; and which is underwritten by the coevolution of musemes with vocal- and rhythmic-production mechanisms, and associated perceptual-cognitive schemata.

## 1 Introduction: music and/as evolution

Progress continues to be made in understanding music's role in human evolution. The evolution of our species, and music's contribution to it, is increasingly well understood, both from a human-centric perspective and one that compares our attributes with those of other species. What appears a particularly promising area of research is the relationship between musicality and music—i.e., between the several biologically evolved structures and competences underpinning the ability to make music (each of which may have been adapted or exapted independently of the others); and the phenomena those structures and competences support and that, as what we now term music, have been subject to cultural evolution over the course of our history (Morley, 2013). Music might itself be subdivided into those processes and phenomena occurring within the boundaries of a work (synchronically) or of a (re-)creative act (diachronically) [Meyer's (1996, p. 24) level of “intraopus style”]; and those occurring without [“extraopus style,” Meyer's (1996, p. 23) level of “dialect”, referring to chronological or geographical style-categories], the latter being the primary site for cultural evolution. Having isolated the two domains of musicality and music [and with a nod to their intra- to extra-opus interconnection, in the form of musicking (Small, 1998)], there seems much promise in research that explores how they relate to each other: that is (perhaps most obviously), how musicality shapes music (nature driving culture); and, conversely (and perhaps more controversially) if and how music shapes musicality (culture driving nature). This article represents a very small contribution to this topic.

To address it, I adopt the perspective of memetics (Dawkins, 1989; Blackmore, 1999; Jan, 2007, 2022; Dennett, 2017) here, in an analysis of ABBA's song “The Winner Takes It All” (ABBA, 1980b)—hereafter “TWTIA” in the main text—in order to understand relationships between the song and another piece of music with which it has certain close connections, namely the love duet “Mon cœur s'ouvre à ta voix”—hereafter “MC” in the main text—the sensuous heart of Saint-Saëns' opera *Samson et Dalila* (1877). In doing this I am retracing, to some extent, the steps of Jan (2003), which similarly considers collections of *musemes* in different musical contexts. The latter are memes in music, being discrete, replicated parcels of music-cultural information^1^ that constitute the building blocks of composed and improvised musics and that, while independently replicated, are often co-replicated as structural-sequential complexes or *musemeplexes*. Some of the connections considered here are quite general, such that, by themselves, they would not warrant our regarding the two pieces as meaningfully related on their account. But another connection—the musemeplex itself—is very specific, and acts as a lodestone, drawing the other connections around it and, thereby, focusing and intensifying their relatedness. This memetic analysis is intended to serve as evidence for my main thesis; that is, *the connections between these two pieces—which are palpable while also elusive—arguably testify to the capacity of two specific categories of museme, namely those corresponding to vocal/melodic and instrumental/rhythmic, to leverage perception, cognition and embodiment in the service of their replication*. Thus, this study is, most tangibly, an exercise in memetic analysis and in the evidencing of the memetic paradigm more generally, as well as—via correlation of these two pieces' memetic attributes with their perceptual-cognitive salience and their image-schematic/embodied aspects—a first attempt to support the afore-stated claim.

To these ends, Section 2 will frame TWTIA in terms of the notion of influence, recast as memetic replication; and it will frame the song as replicating musemes, arranged into a musemeplex, from MC. Section 3 introduces a further issue, namely the distinction between musemes optimized for vocal production and those optimized for instrumental execution. Section 4 gives an overview of the music of TWTIA, considering it in terms of the two main types of music in the song, namely that of the Introduction (hereafter “Intro”)/Chorus/Conclusion (hereafter “Outro”), and that of the Verse. Section 5 compares TWTIA with MC to evidence the claim of memetic replication from the latter to the former. Section 6 uses Narmour's (1990, 1992) Implication-Realisation theory to consider what aspects of the musemes considered contribute to their perceptual-cognitive salience, vis-à-vis the other musemes to which the composers discussed here might also have been exposed. Section 7 assesses how embodiment relates to the musemes that, in previous sections, had been considered primarily in terms of their manifestations in musical notation and sound. Finally, Section 8 aims to draw together the disparate issues covered, attempting to stress the importance of vocality and rhythmicity in memetic transmission when understood in the light of the perceptual-cognitive, image-schematic, and embodied attributes of the musemes replicated.

## 2 Why memetics?

As with all music, and for all its singularity, TWTIA draws upon various influences—different types of borrowings from earlier musical styles. Some of these are quite generic, such as the tonal-harmonic-melodic language, and its bread-and-butter building-blocks, which was normative in “classical” music during the common-practice (Bach-to-Brahms) period and which, despite the radical changes that transformed the language of art music in the twentieth century, remains the basis of much contemporary popular music. Other influences, however, are more specific, and when connections beyond a certain degree of specificity link two pieces of music, it is tempting to ask what might have motivated them, and to ask what they tell us about the relationships between music in seemingly different styles and genres.

To establish some theoretical foundations for discussion of this issue, the concept of influence is often invoked in discussions of “classical” music, with musicologists keen to understand the stylistic relationships between *works*^2^ (Meyer's intraopus style, mentioned in Section 1). Such work-level influences may be, in part, symptomatic of stylistic relationships—perhaps teacher-pupil or peer-peer—between *composers* [Meyer's (1996, p. 24) level of “idiom”]. If the latter relationships span wider expanses of geography or chronology, they are often discussed in terms of connections between wider *styles* (Meyer's dialect, mentioned in Section 1). In addition to considering influence “vertically,” in terms of this hierarchic view of style, others have framed it “horizontally,” as a continuum, ranging—in Cope (2003) model—from long “quotations” to short, generic “commonalities.” The latter are short, widely-used “scraps” of musical material that transcend dialects. They are examples of Dennett's evolutionary “good tricks” (Dennett, 1995, p. 77–78; see also Jan, 2014), which are effective, general-purpose solutions to common design problems, reinvented or seized from an amorphous stock of veridical exemplars (Schubert and Pearce, 2016). Beyond these quite literalistic formulations, which often frame influence as a positive process of absorption, assimilation, and cross-fertilisation, some commentators inclined to a postmodern view would argue that influence—or “intertextuality,” as they often style it (Klein, 2005), meaning connections between works resulting from similarity of material—can also be negative (that which is *not* copied may also be significant); indeed, it can be the source (in Harold Bloom's formulation) of “anxiety” (Korsyn, 1991), or a fear that a work is too similar to its antecedents.

Memetics takes the notion of influence and naturalizes it, seeing stylistic similarities between musical works as the result of the *replication* of some aspect of a source composition in a target work. This replication, memetics hypothesizes, is the consequence of the transmission of some type of patterning—a meme or, in the case of music, a museme—from the brain of one composer to that of another via some intermediate representation, in ways that are analogous to the transmission of the gene in biological replication. In the latter replicator, the *genome* of one organism contributes to that of its descendants via an intermediate *phenotypic* “vehicle,” a physical body or that body's behavior plus any artifacts resulting from the behavior (Jan, 2007, p. 30, Tab. 2.1). In the case of the m(us)eme, and to invent cultural neologisms on the basis of the biological terminology, a *memome* is reconstituted in another brain via the intercession of a *phemotypic* vehicle such as a score or a (live or recorded) performance. Thus, the model for memetics is Darwinism, which arguably operates in a substrate-neutral/agnostic way. That is, nature [including physics (Vanchurin, 2020) and several domains of biology] and culture are hypothesized to be governed by the same fundamental, indeed universal (Dawkins, 1983; Plotkin, 2010), principles of the “evolutionary algorithm” and its three foundational operations of variation, replication and selection (Dennett, 2017, p. 43–47).

To establish the concept of the museme is to differentiate between types of meme by distinguishing between categories of patterns that, while they all behave according to the constraints of Universal Darwinism, nevertheless possess different, substrate-specific properties. Thus, a museme is a short replicated segment of musical information made up (in its phemotypic manifestation) of sound waves that arise from brain structures (in its memotypic incarnation) that privilege—to reduce linguistics to an over-simplified trinity—the phonetic and syntactic domains over the semantic. In notated form, musemes generally align with Miller's (1956) range of 5–9 elements (notes), the lower end (and sometimes below 5) conforming to Cope's (2003) commonalities, the upper (and sometimes above 9) to his quotations. At the risk of conceptual inflation, one might also say that *lexemes* and *sonemes* are similar to musemes, except that the former constitute the sound-patterns of language, and thus privilege the syntactic and semantic over the phonetic (Jan, 2022, p. 10, note 6); while the latter constitute the sound-patterns of animal vocalisations and thus, in perhaps most species, privilege the phonetic and semantic over the syntactic (Jan, 2022, p. 393). *Gestemes*—replicated patterns of gestures (Jan, 2022, p. 197)—have a syntactic and semantic, but not a phonetic component; while *graphemes*—replicated patterns of written symbols (Jan, 2022, p. 91)—echo this but in two, not three, dimensions, and leave a material trace on paper or in other media. The former are, of course, closely connected with the latter, in that a gesteme is necessary in order to give rise to a grapheme. Moreover, the phemotypic products of musemes, such as scores and performances, rely upon gestemes to mediate the necessary physical movements that inscribe (notate) the grapheme-phemotypes that in turn describe and prescribe structure and performative actions. Another category of movement-replicator, *choreoemes* (Jan, 2022, p. 108), perhaps best understood as a sub-category of gestemes, are coordinated sequences of movements that possess the capacity to be understood as choreographic, and thus as potentially artistic and communicative. Yet choreoemes are also likely to be evolutionarily very ancient, given the importance, discussed below, of coordinated social dance to early hominin survival. All of these various replicator phemotypes are coded for by networks of neuronal wiring that, while located in different regions of the brain and interconnected differently with other brain regions (as part of the underpinning of phonetics, syntax, semantics and motor action), nevertheless follow certain common principles of structural organisation (Calvin, 1998). Seen in this way, the notion of the “X-eme”—a replicator in a specific cultural-evolutionary realm—offers a means of unifying phenomena in different domains and, in the case of replicators whose phemotypes include sound-patterns, it helps to mediate between the elements of human music and language and those of non-human animal vocalisations such as the songs of certain birds and cetaceans.

## 3 Evolution and the distinction between vocal and instrumental idioms

If it is accepted that the first music—likely a form of “musilanguage” (Brown, 2000; Mithen, 2006) was vocal—then instruments represent a prosthetic extension of the human (and perhaps non-human-animal) musical body. Initially such instruments—various reed and bone flutes (Conard et al., 2009); external aerophones in the categorisation system of Hornbostel and Sachs (1914)—might be hypothesized to have emulated the prosody of the musilinguistic voice, tracing smooth, *conjunct* (step-wise) melodic motion while being subject to similar constraints of lung capacity as the singing voice of their players.^3^ The nature of instrumental music from its earliest notated forms suggests that, at some point, humans realized that instruments initially developed to emulate the voice were not constrained to that purpose.^4^ Instrument-players determined that, in place of conjunct motion, various *disjunct* (skip-wise)^5^ patterns could be played, this determination perhaps being facilitated by the visual-spatial affordances of such instruments as the lyre, the harp, and the marimba. And they discovered that figures and patterns that would challenge the human vocal performer were readily playable on instruments. Moving beyond the wind instruments built as simulacra of the human voice, other instruments, such as those utilizing keyboards, further leveraged the optimizing power of mechanisms in order to render possible ever more complex and dazzling passagework. Such mechanisms were also added to wind instruments, in order to expedite the use of fingers over the holes deployed to modulate the fundamental pitch of the instrument. Finger holes are evident in the earliest wind instruments, but some of these are perhaps found (Kunej and Turk, 2000), rather than designed (Conard et al., 2009), objects, even though the former attribute does not preclude their musical exaptation.

Of course the foregoing paragraph should not be taken to imply that instruments evolved *exclusively* to emulate the prosody of the human voice. As suggested by the reference above to “prosthetic extension[s] of the human (and perhaps non-human-animal) musical body,” and to the notion of mechanical complexity, instruments can capture both voice-driven melody *and* limb-driven rhythm. Substrates for the latter—underpinning the group rhythmic synchrony in dance likely to have been key to human survival (Merker, 2000; Merchant et al., 2018; Savage et al., 2021)—appear to have evolved separately from (and perhaps before) those for the former—underpinning fine control of F_0 and its hypothesized use in musilanguage, sexual selection (Miller, 2000; Mosing et al., 2014; Ravignani, 2018), and infant nurturing (Dissanayake, 2012). Thus, melodic instruments were at some stage complemented (having perhaps been preceded) by percussion instruments—various wooden and stone drums; struck idiophones in Hornbostel and Sachs (1914)—these designed (or found) to augment and support a regular tactus in communal dancing (and singing) contexts. Percussion instruments might also be regarded as extensions, or imitations, of primate chest-beating behaviors (Salmi and Muñoz, 2020). Of course, melody can also possess a rhythmic dimension, especially when (in a three-note, up-down pattern, for example) a leap upwards or downwards is followed in zig-zag fashion by a return to the vicinity of the original pitch. In such “Reversals,” in Narmour's (1990, 1992) terms (discussed in Section 6), the binarism of the pitch 1-↑-pitch 2 : pitch 2-↓-pitch 3 creates the impression of a pendulum-like impulse, with all the potential for measured rhythmic continuation—in a musical equivalent to bipedalism—this implies. In short, melodic and percussion instruments are the epiphenomena of complex biological- and cultural-evolutionary processes that interconnect fine vocal control and vocal learning (Merker, 2012) with rhythmic entrainment and group synchrony (Brown, 2022).

From this complex evolution arises the distinction between what Ratner (1980, p. 19–20) terms the “singing style” and the “brilliant style” in late-eighteenth century European music, one prefigured by Mattheson in his *Der vollkommene Kappelmeister* of 1,739 (Mattheson and Harriss, 1981), who argued for the aesthetic superiority of vocal music over instrumental music. The former style refers to the conjunct, narrow-tessitura (vocal range), even-rhythm organisation, and measured pace generally regarded as idiomatic in music for voices; the latter to the often disjunct, wide-tessitura, uneven-rhythm organisation and faster pace generally typical of idiomatic music for many instruments. By invoking the notion of style, Ratner is keen to stress that these two “topics” (Mirka, 2014) are not confined to voices and instruments, respectively. Rather, the singing style can also be adopted in instrumental music;^6^ and, conversely, the brilliant style can also be adopted in vocal music. Examples of the former include the slow movement of Mozart's Clarinet Concerto K. 622 (indeed, the slow movements of most classical-period concerti, irrespective of the solo instrument); examples of the latter, from the same composer, include the Queen of the Night's arias in *Die Zauberflöte* K. 620, both from 1791.^7^

While the aforementioned properties of the singing and brilliant styles are typical, it is the case that—because notions such as narrow/wide and measured/rapid exist on a continuum—they are not clear-cut categories. Indeed, the two styles inevitably manifest some overlaps, in these and in other aspects. In particular, there are situations in vocal/singing-style music where there is a leap, just as there is often step-wise motion in instrumental/brilliant-style music (in fast scalic passages, for example). While certain intervals, such as augmented fourths/diminished fifths, sevenths and ninths, are rare in vocal leaps (certainly in most tonal vocal music), other larger intervals, such as perfect fourths and fifths, and sixths are more common. As noted above, very often a skip is followed by step-wise motion, generally in the opposite direction, so as partially to “fill-in” the skip—a “gap-fill” motion, in Meyer's terms (Meyer, 1973; see also Bigand, 1993). Such Narmourean Reversals satisfy a perceptual-cognitive urge that has its basis in an evolutionary-physiological constraint (Matzinger and Fitch, 2021). As formalized in Fux (1965) on the basis of the style of Palestrina (Jeppesen, 1992), a skip in one direction followed by motion, step or skip, in the same direction will risk moving outside the natural range of the voice, so a reversal is in part a tessitura-preserving mechanism, one that persists even in instrumental music, where there is normally a wider tessitura available, as an artifact of the vocal origins of human music (see also Huron, 2001). Nevertheless, when a vocal line incorporates a wide leap, this might be regarded as an incursion, however fleeting, of the brilliant style; and, conversely, when an instrumental line adopts conjunct motion, this might be regarded as the influence, again perhaps limited, of the singing style.

From Ratner's distinction between the singing and brilliant styles, one can move to the notion of embodiment. An element of the “4E”—embodied, embedded, enacted, extended—model of cognition (Newen et al., 2018; see also Carney, 2020), this is the idea that, as well as processing music cerebrally, as a sequence of abstract mental representations, humans also experience it viscerally, in terms of the physical movements it impels and recalls (Leman, 2008; Shapiro, 2011; Cox, 2016; van der Schyff et al., 2022). Indeed, in many cultures, music and dance are performatively, and often linguistically, inseparable. Embodiment draws upon the notion of image schemata (Spitzer, 2004; Snyder, 2009), whereby positional location in three-dimensional space, direction and velocity of travel, and size, are felt in diachronic sound-streams as motivations to move the body and to imagine the music as itself moving as a living agent would. Given their likely separate evolutionary trajectories, it seems the case that vocal music (and instrumental music adopting the singing style) is embodied differently to instrumental music (and vocal music adopting the brilliant style). Of course, embodiment is not a tangible quantity, measurable according to some metric; rather, it is a subjective attribute whose intensity varies even in the same individual according to a number of factors. Nevertheless, and to restate the “main thesis” outlined in Section 1, if it is the case that there is a different kind of embodied physicality attendant upon vocal music and/or singing-style music, as compared with instrumental music and/or brilliant-style music—i.e., that the former draws upon primarily vocal modes of line, continuation and *telos*; whereas the latter draws upon the iterative, cyclic and cumulative—then this distinction would appear to have clear cultural-evolutionary implications for those types of music (insofar as they are separable).

Specifically, and to expand upon the claim articulated in Section 1, one might hypothesize that musemes that conform to the singing style achieve their fecundity (the result of perceptual-cognitive salience) in ways that are different from musemes that conform to the brilliant style. While this is partly a function of different modes of memorability—a slow, conjunct passage is likely to recruit different strategies for memorisation than a fast, disjunct one—it is also a consequence of the power of vocal- versus instrumental-style musemes to leverage perception, cognition and embodiment in different ways. Put simply, vocal-style music (whether delivered by voices or instruments) is differentially more affective, and instrumental-style music (whether delivered by instruments or voices) is differentially more kinetic, because they connect in different ways with our morphology, our physiology and our psychology, as shaped by millennia of gene-meme coevolution (Azumagakito et al., 2018). By virtue of this connection, music characterized by musemes in one (or both) of these categories is also likely to motivate gestural patterns that align with and reinforce the evolutionary narrative of its category-specific musemes.^8^

## 4 Musemes in “The Winner Takes It All”

TWTIA is the first song in ABBA's album, *Super Trouper* (ABBA, 1980a,b), with it and the album's title song achieving number 1 in the charts. It occupies a central position in the musical *Mamma Mia!* (Andersson et al., 1999), subsequently made into a film (Lloyd, 2008) in which its prominence is further heightened by its position in the climactic scene. In many ways the song represents the apotheosis of the group's fame and fortune and is arguably a *locus classicus* of their style. Moreover, its lyrics are a commentary on the break-up, in 1979, of one of the two couples constituting ABBA, Agnetha Fältskog and Björn Ulvaeus; the other couple, Anni-Frid Lyngstad and Benny Andersson, were themselves to separate in 1981.

Given the point made in footnote 2 about prescriptive *versus* descriptive notation, scores of this song are often transcriptions (many poor-quality versions are available on the internet, often simplified for the benefit of beginners). The version used here, however (Andersson and Ulvaeus, 1999, p. 107–111), is that from the official “vocal selections” book of the musical, issued, presumably, under Andersson's and Ulvaeus's imprimatur. I focus principally on this printed score, as opposed to the recording, because the score is the most straightforward means of illustrating the connections that I regard as central here. This is certainly not to downplay the importance of the recording (ABBA, 1980a), which is arguably not only the song's definitive version, but also its fundamental ontological state (Nattiez, 1990, p. 69), and which may itself serve as the object of an analysis (Cook et al., 2009). As a result of this focus on the vocal selections score, and for reasons of space, I am also neglecting consideration of the bass guitar, acoustic guitar, and massed backing vocals. While there is an inevitable loss in terms of discounting the textural and timbral richness of the song,^9^ the vocal selections score gives all the necessary melodic and harmonic information for present purposes.^10^

TWTIA is a strophic song with four verses, each of which is followed by a chorus, these eight sections being bookended by an Intro and Outro. The repetition-structure of the vocal-selections score is complex owing to the number of time-bars and the “To Coda ⊕” sign (Andersson and Ulvaeus, 1999, p. 109) after Verse 3—which, on my interpretation, takes one *first* to Chorus 4 and *then* to the coda. There are only sixteen unique bars of music in TWTIA, the song achieving its length by skilful repetition of these bars. One group of eight form the material (variously disposed and orchestrated) of the Intro, the Chorus, and the Outro, discussed in Section 4.1; and a second group of eight form the material of the Verse, discussed in Section 4.2. Within these sections, their total length of sixteen bars each—Chorus 1 and Chorus 4 are, however, only eight bars in length—is achieved by repetition of the “Intro/Chorus/Outro-eight” phrase and the “Verse-eight” phrase.

### 4.1 Intro/Chorus/Outro

**Figure 1:**
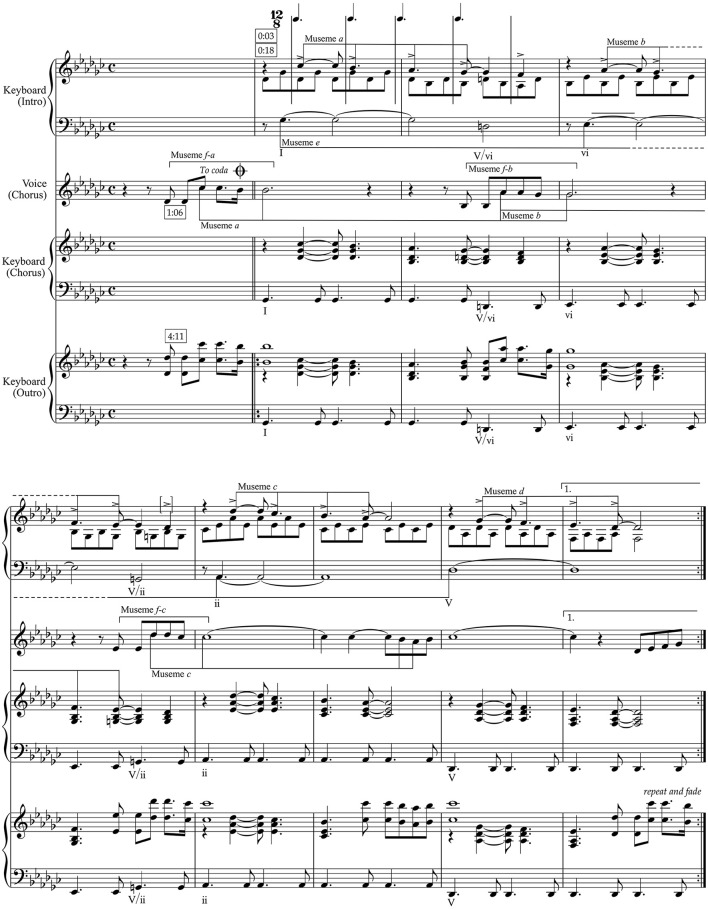
Intro/Chorus/Outro music in “The Winner Takes It All”.

Figure 1 shows the Intro/Chorus/Outro music. Boxed time-codes above the score refer to the corresponding points in the official video (ABBA, 1980b), available at https://www.youtube.com/watch?v=92cwKCU8Z5c, and allow the score to be followed in conjunction with the video.

**Figure 2:**
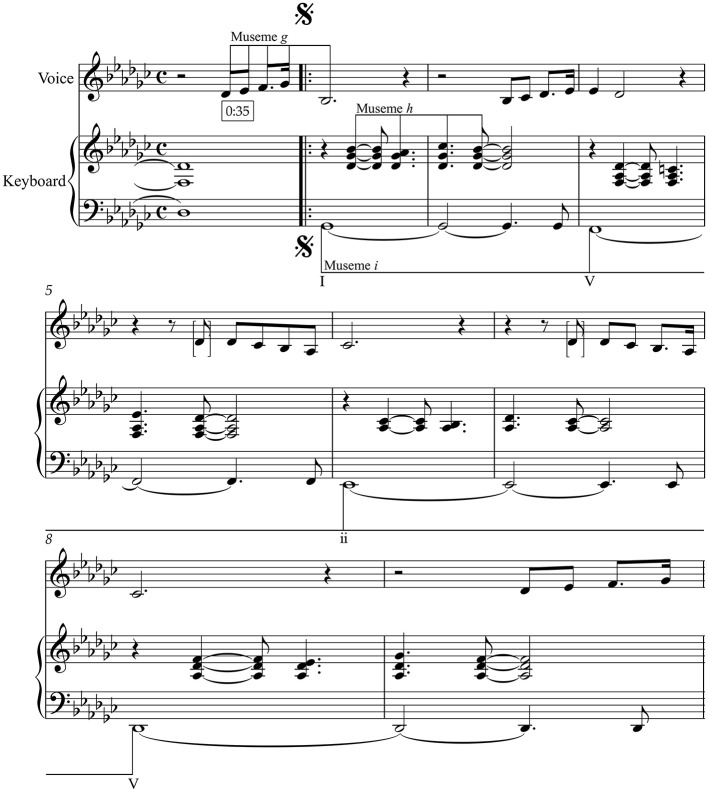
Verse music in “The Winner Takes It All”.

This music is based on a standard chord progression in tonal music, I–vi–ii–V, with the second and third of these preceded by “secondary-dominant” harmonies (V/vi and V/ii, respectively) that temporarily tonicise them.^11^ The I–vi–ii–V sequence is labeled “Museme *e*” (hereafter in the text “M*e*”, etc.) in Figure 1, because it is a replicated pattern in tonal music, and appears not only in this song but in numerous other contexts. Its initial I–vi progression is itself a commonplace of tonal harmony—it is an independent museme, embedded within and constituting part of a longer one, M*e*—and is often used in popular music to illuminate two sides of the same emotional coin, often the pleasures and pains of love (in this case remembered). Musemes are labeled alphabetically and consecutively in Figure 1, this labeling continuing into Figure 2, starting from the first bar of the upper stave which, as noted below, is the beginning of M*a*. It will be understood from Figures 1, 2 that nine musemes—M*a*–M*i*—make up the bulk of the material of this song, arranged in strata of different duration from the short segments of M*a*–M*d* and M*f* –M*h* to the longer, section-underpinning M*e* and M*i*.^12^

Melodically, the Intro/Chorus/Outro has a very characteristic stylistic feature of ABBA, the appoggiatura,^13^ given extra salience here by the accent, which compensates (in the case of the first instance, b. 1^2^, and comparable cases) for the weak-beat rhythmic placement.^14^ The two appoggiaturas and their resolutions spanning bb. 1–2 outline the scale-degree progression $\hat{4}$–$\hat{3}$–$\hat{2}$–$\hat{1}$ (M*a*), which is continued over the next six (3 × 2) bars as $\hat{2}$–$\hat{1}$–$\hat{7}$–$\hat{6}$ (bb. 3–4; M*b*), $\hat{5}$–$\hat{4}$–$\hat{3}$–$\hat{2}$ (bb. 5–6; M*c*), and $\hat{1}$–$\hat{7}$–$\hat{6}$–$\hat{5}$ (bb. 7–8; M*d*).^15^ Calculating the scale-degree sequences of the second, third, and fourth two-bar phrases in terms of the “tonic” of their associated harmony (vi, ii, and V, respectively) renders them all as (locally) $\hat{4}$–$\hat{3}$–$\hat{2}$–$\hat{1}$, so bb. 3–8 constitute three sequential repetitions of bb. 1–2. Nevertheless, while essentially the same *tetrachordal* (four-note) pattern, they are labeled as separate musemes on account of their distinctive pitch, scale-degree, and intervallic structures, as well as their subtly but distinctly differing ranges (greater vocal effort is required, for instance, to execute M*c* compared to M*a*). All three of these criteria are important for defining musemes, and will be drawn upon in this article. While scale-degree structure is arguably primary (given the tendency for imitation of patterns often to occur in different keys), there are situations where absolute pitch is also significant. Note also that M*a* and M*b* are extended beyond the tetrachord of M*c* and M*d* to accommodate melodically the secondary-dominant harmonies below them (which do not occur in conjunction with M*c* and M*d*). For the purposes of this article, these extensions (to f^1^ in b. 2^4^ and to d♭^1^ in b. 4^4^) are not regarded as integral to the musemes.^16^ They might be regarded as intersecting with a different family of musemes—a *museme allele-class* (Jan, 2016), or set of structurally/functionally similar musemes, or *museme alleles*; discussed further in Section 5—the secondary-dominant harmonies being instances of the kinds of musical forces that, in mutating extant musemes, engender new forms and new encompassing allele-classes. These differences between M*a* and M*b*, on the one hand, and M*c* and M*d*, on the other, contribute to the sense that the division of these eight bars into four groups of two bars and two groups of four bars creates motion in the first group of four bars and a move toward arrival in the second group. In terms of the singing/brilliant-style distinction, however, M*a*–M*d* are all clear examples of the former.

The first of the secondary-dominant harmonies, the V/vi of b. 2^3–4^, sounds under the final g♭^1^, b. 2^3^, of M*a*, creating a brief augmented triad (B♭ –D𝄮 –G♭), an unstable tonal aggregate that impels the melody line to resolve down to the f^1^ of b. 2^4^. In this way a stable four-note museme, M*a*, is destabilized and resolves to a note that creates a five-note museme. A similar process occurs in b. 4^3–4^, except the V/ii does not create an augmented triad, and so the movement of the melody line from the e♭^1^ of b. 4^3^ to the d♭^1^ of b. 4^4^, while it still creates a five-note line, and while it anticipates the following d♭^2^ (b. 5^2^) of M*c*, is not as strongly motivated. The remaining musemes here, M*c* and M*d*, do not have this extension, owing to the stability of their supporting harmonies (ii, V, respectively). The harmonic support, consonance-dissonance structure, and analogy to M*c* and M*d* establish M*a* and M*b* as four-note musemes, but the extension, particularly of M*a*, is relevant from the perspective of museme salience.

In its Chorus-incarnation, this eight-bar phrase retains the same harmonic framework but the initial appoggiaturas of M*a*–M*c* are prefixed in the vocal part by a distinctive upbeat-functioning museme of two repeated notes followed by a rising interval of a seventh that forms the bulk of the vocal line in the Chorus. Its iterations are marked M*f-a*, M*f-b*, and M*f-c* in Figure 1 because the upbeat rising-seventh museme, labeled M*f*, is associated with the following statements of M*a*, M*b*, and M*c*, respectively. The M*f-a/b/c* musemes link the four two-bar units of the Chorus phrase together and—because M*f-a* occurs at the end of the previous Verse, over its end-V^7^ chord—it makes M*a*, M*b*, and M*c*, originally primary in the Intro, sound retrospectively like echoes of M*f-a*, M*f-b*, and M*f-c*, respectively. The rising seventh interval of M*f-a*, M*f-b*, and M*f-c* represents a deviation from the conjunct lines of M*a*–M*d*, despite its association with the first three of these musemes. In this sense, and as suggested in Section 3, it represents an incursion of brilliant style—the seventh is traditionally not regarded as a smooth or easy interval to sing—into the singing style previously associated with TWTIA's melody.

### 4.2 Verse

Figure 2 shows the Verse music. Again, boxed time-codes above the score refer to the corresponding points in the official video (ABBA, 1980b).

The melody line of the Verse uses the tetrachordal shape of M*a*, M*b*, M*c*, and M*d* from the Introduction/Chorus/Outro material to create an opening museme labeled M*g* on Figure 2. This, it will be understood, is a retrograde (a backwards-version) of the M*a*–M*d* tetrachord—specifically the pitch-sequence of M*d*—ending with a distinctive falling sixth from g♭^1^ to b♭. In what may be a further mutation of the tetrachord pattern—or a member of a different museme allele-class—the falling-fourth line of M*a*–M*d* is reworked into a “changing-note” figure of a type discussed in literature on eighteenth-century “galant” figuration^17^ and labeled M*h* here.^18^ Numerous such figures exist, but M*h* characteristically departs from, then returns to, its initial pitch. While these patterns—conceived as cognitive schemata by Meyer and Gjerdingen—are usually associated with changing harmonies (generally I–V …V–I) in 18th-century music, in the song the four iterations of M*h* are each presented over a single bass note and unchanging right-hand harmony.

In a third iteration of a tetrachordal museme in this section, the bass line, labeled M*i*, falls from G♭ to D♭ ($\hat{1}$–$\hat{5}$), bookending the phrase with tonic and dominant harmonies, respectively. Although labeled M*i*, it is identical in pitch and scale-degree sequence to M*d*: the former is four times the duration of the latter as a consequence of being a quarter the “speed,” indicating that essentially the same museme can operate at different metric-hierarchical/levels. Such falling tetrachordal bass lines have a long history in tonal music in both the major- and (the various) minor-key forms, and they were widely assimilated, particularly in the major-key form, in popular music from the 1950s onwards. They are a subset of bass lines that traverse a larger interval, often in the context of repetition, as in the chaconne and passacaglia types. The major-mode form of the tetrachord bass often connote images of eternity and stasis—owing in part to their use of cyclic processes (McClary, 2004)—as in, for instance, Procol Harum's “A Whiter Shade of Pale” (Brooker et al., 1967), with its clouded allusions to J.S. Bach's “Air” from the Orchestral Suite in D, BWV 1068 (*c*. 1730). Minor-mode incarnations of the tetrachordal bass lines, by contrast, are often associated with darker emotions, and in this mode they are apt to employ the fully chromatic form, which uses an admixture of the two versions (ascending and descending) of the melodic minor scale (Williams, 1998), as in Dido's lament “When I am laid in earth” from Purcell's opera *Dido and Aeneas* (1689). Such associations of musemes with “verbal-conceptual” memes—here articulating notions of sadness and death—is essentially the basis of evolutionary cultural semiotics (Koch, 1986). The tetrachordal musemes of the Introduction/Chorus/Outro and Verse are discussed further in Section 6.

## 5 Connections with “Mon cœur s'ouvre à ta voix”

It might seem odd to posit a connection between a duet from a French Grand Opera of the 1870s and a popular song of the 1980s.^19^ There are, nevertheless, a number of generic connections, one quite specific, that tie MC and TWTIA together closely. Moreover, there is what might be termed a potential intermediate vector linking the opera with ABBA's song, namely the rhythm and blues singer Jackie Wilson's song “Night” (Saint-Saëns et al., 1960), which is essentially an arrangement of Saint-Saëns aria, specifically the sections referred to below as B^1^ and B^2^. It is highly likely that the composers of TWTIA would have known this song: Andersson was fourteen when it was released in 1960 and Ulvaeus was fifteen; and if they did not encounter it on its initial release, they would almost certainly have heard it at some point during the 1960s, given its continuing popularity (this perhaps heightened by reporting of Wilson's colorful private life). It may also have been the case that the centenary of the opera—in 1977, also the year in which one of the greatest interpreters of the role of Dalila, Maria Callas, died—was celebrated (or at least marked by an increased number of performances) in ways that might have come to the attention of the members of ABBA.^20^ It should be stressed that the connections outlined below inhere in musical, not textual, relationships: as Appendixes 1 and 3 indicate, there are few concrete similarities between these pieces at a lyrical/narrative level beyond their female subject-position.

**Figure 3:**
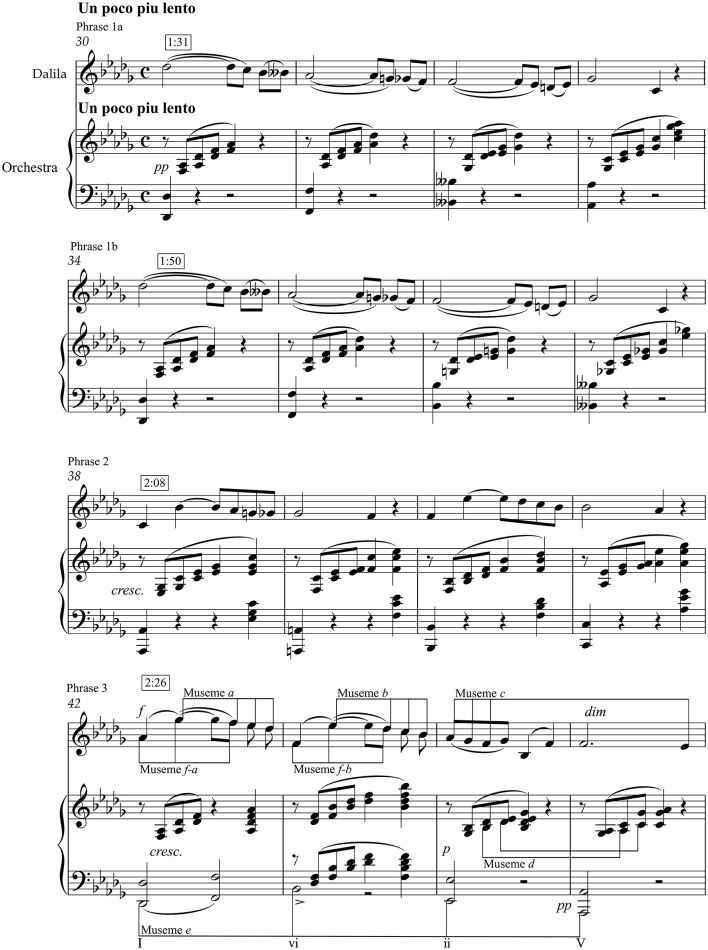
Saint-Saëns: “Mon cœur s'ouvre à ta voix”, Section B.

At the most general level, MC might be regarded, like TWTIA, as broadly strophic, in that it contains verse-like, dynamic/narrative text, and chorus-like, static/contemplative text. This is associated with two types of material: the opening *Andantino* 34-time music, and the subsequent *Un poco più lento* 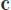-time music, the latter shown in Figure 3, which alternate in an A^1^ (bb. 1–29)–B^1^ (bb. 30–49)–A^2^ (bb. 50–78)–B^2^ (bb. 79–100) sequence. Comparing the music of TWTIA with that of MC, and while there are a number of aspects that relate the two pieces, the most striking connection between MC and TWTIA—the “lodestone” referred to in Section 1—concerns the passage shown in the bottom system of Figure 3, the climax of the B^1^/B^2^ sections of MC, which I term Phrase 3, bb. 42–45 in B^1^, repeated in bb. 46–49; Phrase 1a is bb. 30–33, Phrase 1b is bb. 34–37, and Phrase 2 is bb. 38–41. Boxed time-codes above the score refer to the corresponding points in the video of a performance by Elīna Garanča given at the Vienna Opera Ball in 2011 (Saint-Saëns, 2011), Available online at https://www.youtube.com/watch?v=eMadr61Wq_E, and allow the score to be followed in conjunction with the video.

On first glance, this phrase may not appear to bear any resemblance to TWTIA, but it is in fact very close to the Intro/Chorus/Outro music if one takes the chord progression and scale-degree sequence into account. Firstly, the chord progression here—I–vi–ii–V—is identical to that of TWTIA's Intro/Chorus/Outro, albeit without the secondary-dominant harmonies before vi and ii. In addition to this harmonic similarity, Figure 3 also shows that the melodic structure of TWTIA's Intro/Chorus/Outro is also present. Specifically, MC presents M*a*, M*b*, and M*c* in sequence (the first two with the M*f* prefix) and in alignment with the same harmonies as in TWTIA; and it is also possible to read the presence of M*d* if one considers the accompaniment of bb. 44–45.^21^ These connections suggest that Anderson and Ulvaeus were influenced by MC in writing their song—either directly or, via one or more intermediate vectors potentially including the Wilson song, indirectly—replicating the harmonic and melodic musemes of these few bars of MC to create their Intro/Chorus/Outro music.

**Figure 4:**
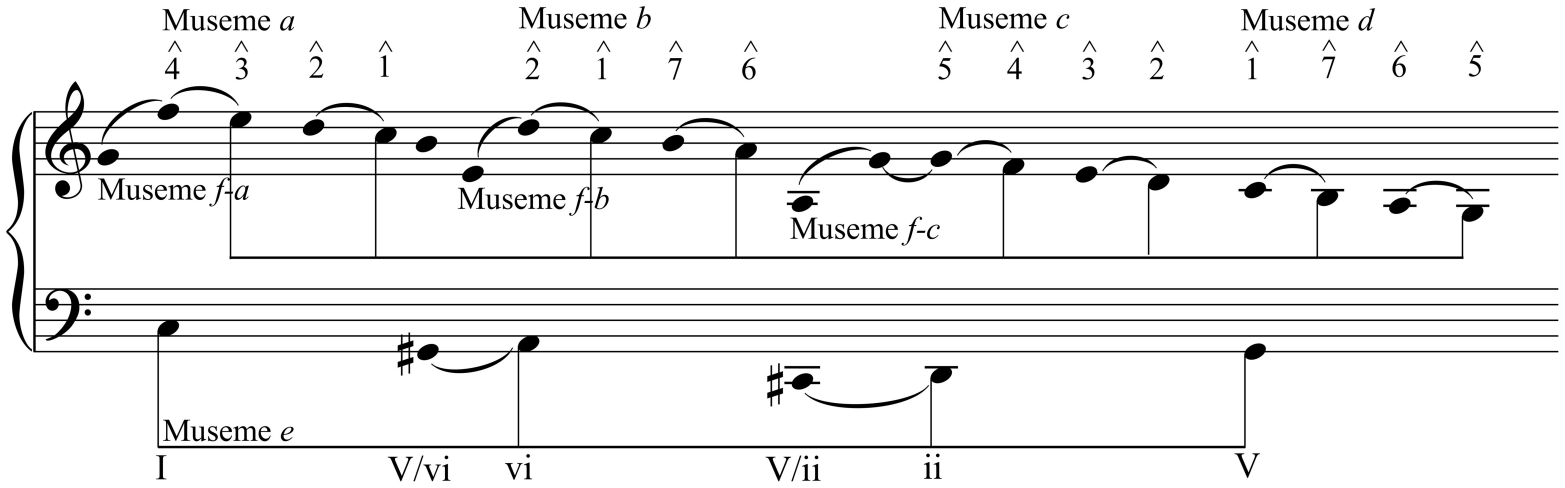
The MC-TWTIA musemeplex.

Figure 4 shows the elements common to both MC and TWTIA, transposed to C major for clarity. It uses a broadly Schenkerian approach—a reductive method of musical analysis that seeks to reveal the underlying framework of a passage or movement by simplification and thereby, more broadly, to represent the underlying tonal grammar of music (Schenker, 1979)—in order to illustrate the close similarities between these sections of the two pieces. Note that Figure 4 rationalizes the register of M*a*–M*d* by conforming to the model of MC, where the pattern is M*a* ↓ M*b* ↓ M*c* ↓ M*d*; in TWTIA, the pattern is M*a* ↓ M*b* ↑ M*c* ↓ M*d*. While this difference is partly a consequence of the different keys of the two pieces—in G♭ major, TWTIA starts in a lower register that the D♭ major MC—it creates a subtly different effect in each: in MC, the expansion of range, and the subsequence uninterrupted descent, gives a sense of space and inevitability; in TWTIA, the contraction of range is associated with the additional intensity afforded by the rise from M*b*–M*c*.

As its caption indicates, Figure 4 shows a musemeplex common to these two pieces. A musemeplex might formally be defined as a collection of two or more musemes that, while replicated independently of each other, are also replicated as a complex. That M*a*–M*e* are independently replicated can be taken as read, given that, as tetrachordal patterns, they are common in all tonal music. That they are replicated as a complex is evident from Figure 4, which shows that they are present in both MC and TWTIA (and possibly also in other music). A logical attribute of a musemeplex is that the collection of musemes that constitute it might be reduced (as can all tonal music) to the underlying framework that underpins it. In Schenkerian terms, reduction of foreground details reveals a (shallow-) middleground structure beneath. This structure—what might be termed a *musemesatz* (Jan, 2022, p. 203)—is common to two or more musemeplexes, whether they replicate the same collection of musemes (a “real” musemeplex), or whether one or more of the musemes in the antecedent musemeplex is replaced by one of its alleles in the consequent musemeplex (a “virtual” musemeplex) (Jan, 2022, p. 202–203). As indicated in Section 4.1, by a museme allele is meant—by analogy with a gene allele—a rival or alternative form of a museme, such that members of a given museme allele-class have sufficient structural and functional similarity that they can occupy a given *locus*—a linear-sequential “slot”—in a musemeplex (Jan, 2010).

## 6 Memetic-evolutionary aspects in the light of Implication-Realisation structure

Up to now this article has considered some of the significant patterning in MC and TWTIA but has not yet framed them in specifically evolutionary terms, beyond asserting that they are musemes. In terms of supporting this assertion, there are two factors that reinforce the claim that the patterns are related in such a way as to warrant the symbolic appellations (M*a*, M*b*, etc.) that infer their memetic-evolutionary relationship.^22^ Firstly, the historical linkages from Saint-Saëns to Andersson and Ulvaeus do not contradict any potential connections; indeed, they positively support them.

Secondly, whereas a two- or three-note commonality might not be regarded as evolutionarily significant—its appearance might be the result of homoplasy, not homology—once one gets to the four-note (M*a*–M*d*, M*e*, M*h*, and M*i*) and five-note (M*f-a/b/c* and M*g*) patterns involved here, then the hypothesis of (conscious or unconscious) transmission becomes progressively more convincing, on information-density grounds. That is, the more pitches that constitute a museme, the more interconnections/relationships that exist between them. Additionally—and while not linear correlations—the higher the information-density, the higher the perceptual-cognitive salience, and the higher what might be termed the transmission-clarity, or the confidence with which a posited antecedent can be connected to its hypothesized consequent (Jan, 2007, p. 61). Moreover, three of the four-note musemes—M*a*, M*b*, and M*c*—are given considerably greater salience by their co-replication with the rising-seventh M*f*, in both MC (M*a* and M*b* only) and TWTIA (M*a*, M*b*, and M*c*). This association is “tight” in MC (Figure 3), but it is “loosened,” and arguably made more prominent, in TWTIA (Figure 1). Finally, and perhaps most significantly, the saliency effects of individual musemes is augmented by their association in a musemeplex. As with other musemeplexes, that in MC and TWTIA (Figure 4) is perceptually-cognitively salient in part by virtue of the number of relationships it encompasses and sustains: it is an information-rich nexus of synergistic interconnections that augments the individual “pointedness” of its component musemes. Such augmentation means that musemes that would individually function as commonalities lose their good-trick attribute, because the likelihood of their having been memetically replicated (as opposed to their having been re-invented or drawn from a generic store of useful figures) is higher, on account of their co-replication with other such musemes in the complex.

If one is prepared to believe that these patterns are indeed musemes, involved in relationships of replication by transmission, then it is necessary to ask what it is about them, beyond the above appeal to information-density, that makes them copyable: what attributes do they possess that make them sufficiently distinctive and memorable that the composers discussed here felt compelled, at some level, to use them in their compositions instead of any other patterns?

**Figure 5:**
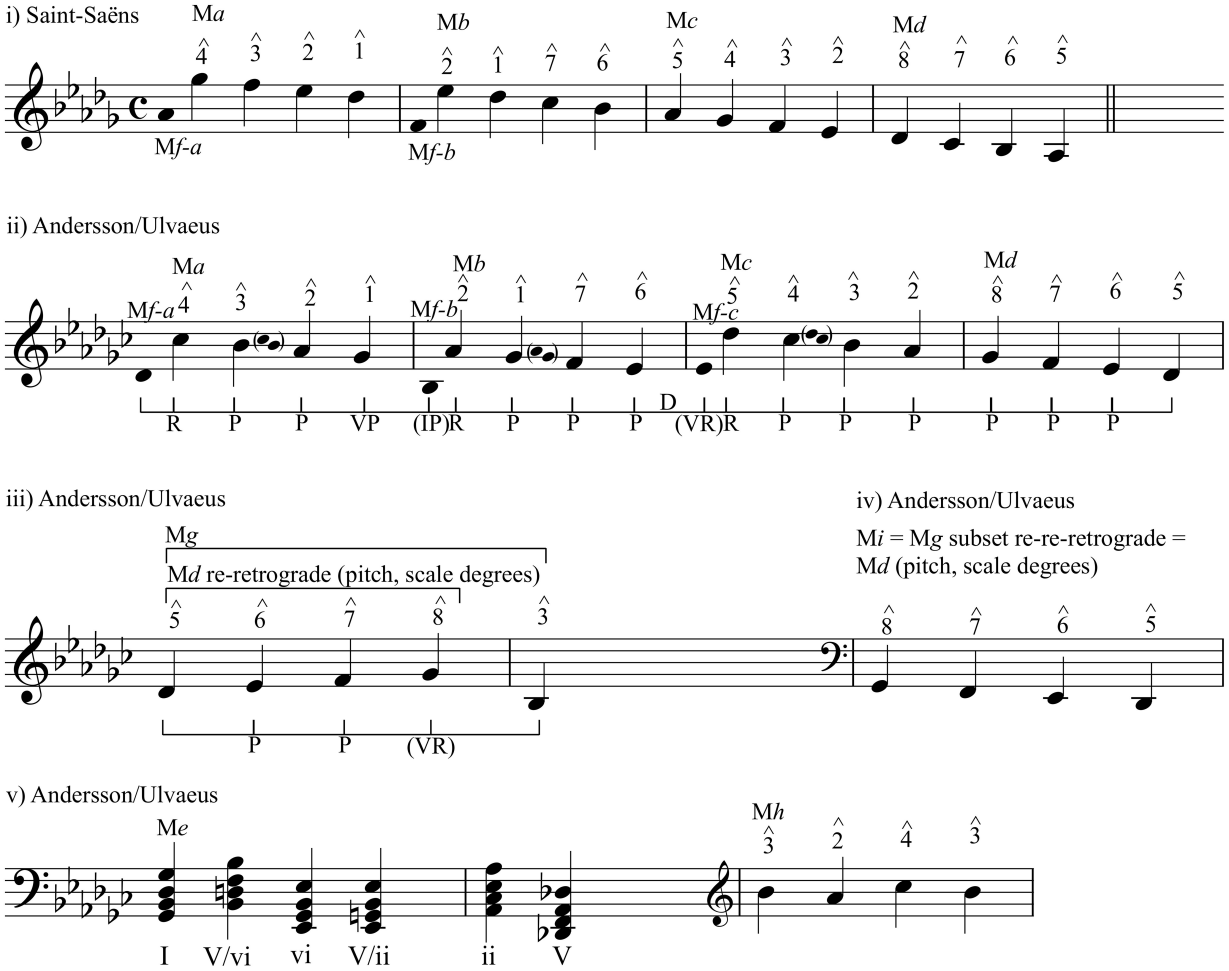
Implication-Realisation structure of M*a*–M*d* and M*g*. P, Process; R, Reversal; VP, Prospective Registral Process; (IP), Retrospective Intervallic Process; D, Duplication; (VR), Retrospective Registral Reversal. See explanations in the main text.

One explanation might draw, in part, on Narmour's Implication-Realisation (I-R) theory, which hypothesizes that musical patterning, once initiated, carries within it certain implications for continuation that, if realized, would create a sense of continuity and openness and, if frustrated, would not only impose closure, but would impart to the museme a heightened “presence” (with respect to similar musemes without this feature).^23^ Depending upon the size of the initial interval, implications inhere in intervallic similarity/dissimilarity, and in registral (directional) continuity/discontinuity. M*a*–M*d*, with their M*f-a*–M*f-c* prefixes in TWTIA's Chorus and Outro, and M*g* in TWTIA's verse, are interesting in this regard, and their I-R structures are represented (by means of the brackets underneath the score marked with various I-R symbols) on Figure 5, (ii). Figure 5 (i) and (ii) show the relationships between M*a*–M*d* in MC and TWTIA. Figure 5 (iii) and (iv) show how the tetrachordal structure of M*d* underpins—by various manipulations—that of M*g* and M*i*. Note that M*a*–M*c* and their prefixes M*f-a*–M*f-c*, respectively, are “telescoped” together in Figure 5 by omitting repeated notes, and that the real I-R structure, described by the interlocking vocal and instrumental lines (and represented in part by the small bracketed notes on Figure 5), is therefore more complex.

The symbols in Figure 5 (ii) and (iii)—representing six of the sixteen I-R structures theorized by Narmour—are explained below:

- “Process,” **P** = after a small interval, a continuation in the same direction with another small interval (Narmour, 1990, p. 89)^24^;
- “Reversal,” **R** = after a large interval, a continuation in the opposite direction with a smaller interval (Narmour, 1990, p. 151);
- “Prospective Registral Process,” **VP** = after a small interval, a continuation in the same direction but with a large interval (Narmour, 1990, p. 330–331);^25^
- “Retrospective Intervallic Process,” **(IP)** = a zig-zag pattern of similarly large intervals (Narmour, 1990, p. 353);^26^
- “Duplication,” **D** = a repetition of a note (Narmour, 1990, p. 97);
- “Retrospective Registral Reversal,” **(VR)** = after a small interval, a continuation in the opposite direction with a large interval (cf. **VP**) (Narmour, 1990, p. 335, Ex. 20.5).

At the risk of tainting Narmour's I-R theory with the Schenkerism it aims to supplant (Narmour, 1977),^27^ the “sub-foreground/shallow middleground” I-R structures of these musemes are summarized below.

1. M*a*–M*d* constitute a double **P**: a three-note **P** interlocks with another three-note **P**, giving a strong sense of downward momentum.
2. M*a*–M*c* are each prefixed with M*f-a*–M*f-c*, respectively, and this figure has significant consequences for the associated I-R structures:
  - The M*f-a*–M*a* juxtaposition creates an initial **R**, before the first **P** of M*a*. This has strong expressive potential, owing to the tension (first interval)-resolution (second interval) curve of the **R** structure.
  - The M*a*–M*f-b*–M*b* juxtaposition creates an interlocking **VP**–**(IP)**–**R** structure. As partial denials of expectation, the **VP**–**(IP)** sequence attenuates the energy of the preceding **P**s, imposing a museme segmentation boundary and a cognitive-expressive “jolt” (Narmour, 1990, p. 138) over the barline.
  - The M*b*–M*f-c*–M*c* juxtaposition creates an interlocking structure of a **P** followed by a **D** (of the e♭^1^ over the bar line), a **(VR)**, and then the familiar **R**. Again, the energy and directionality of the **P**s is counteracted by the contrary force associated with the radical change of direction impelled by the **(VR)**–**R** structure.
  - The M*c*–M*d* sequence is uncomplicated, being a series of interlocking **P**s. This correlates, arguably, with the sentiment of inevitability expressed by the text.
3. M*g* is even more perceptually-cognitively “charged” than M*a*–M*d*, in that the rising tetrachord is conjoined with a falling minor sixth (g♭^1^–b♭) that creates a **(VR)**. Note also that the **(VR)**'s generating f^1^–g♭^1^–b♭ pitch sequence may derive from the **VP** at the end of M*a*—a♭^1^–g♭^1^–b♭ —or perhaps be heard as a musical link.

Discussing these patterns in terms of Narmour's I-R theory is not meant to “prove” that they are connected to each other via memetic-evolutionary replication, but it certainly helps to support an assertion of musemic similarity. That is, two identical musical patterns will, self-evidently, have the same internal I-R structure, as might two very similar patterns. In this sense, the I-R structure either reinforces the arguably more tractable intervallic, pitch, and scale-degree resemblances; or it allows distinctions to be drawn between two superficially similar patterns that, owing to the “categorical” nature of I-R structures (something is, by definition, a structure X or it is not), might nevertheless be distinguished on account of I-R differences, and therefore assigned to a separate museme allele-classes.

What the I-R theory does offer, however, is some means of assessing the salience of musemes, and therefore of distinguishing between the relative salience of two or more musemes (see Jan 2007, p. 130–133 for a proposed quantification) in situations where other factors are relatively consistent. This is beyond the scope of this article to prove, because this research is by nature empirical and statistical: it requires the survey of large bodies of music in order to segment it into its constituent musemes (whereby “that which is copied may serve to define the pattern”; Calvin, 1998, p. 21; see also Meredith, 2016); followed by the comparative analysis of the I-R salience of the resultant musemes (which should correlate with the musemes' relative representation in a cultural community; Jan, 2014, sec. 6). Nevertheless, it is possible in principle that contextual factors—the relationships connecting MC and TWTIA, “primed” by the currency of the Jackie Wilson version—worked synergistically with the perceptual-cognitive salience effects of the I-R patterns, particularly those resulting from the juxtaposition of the isolated tetrachordal “cell” to create the M*f-a*–M*a*–M*f-b*–M*b*–M*f-c*–M*c*–M*d* sequence. These contextual and salience effects may have made the composers of TWTIA more disposed, in a statistical sense, to draw upon MC than they would have done had these conditions been different.

One must take care to distinguish *inter*-work processes from *intra*-work processes. That is, TWTIA's M*g* might exist in the melody of the verse because Andersson and Ulvaeus copied it, directly or indirectly, from some antecedent, in a process of musemic transmission. But it might also have arisen from the intra-work processes of tetrachordal manipulation represented in Figure 5. The latter scenario still counts as a memetic—strictly a mnemonic (Lynch, 1998)—process, because musical patterns are being replicated (albeit from one part of the brain to another) with variation and selection; but the intra-brain recreation of a pattern by means of the variation of notionally intermediate forms does not have the same cultural-evolutionary force as phemotypically mediated inter-brain memetic transmission.

## 7 Music, memes, and embodiment

While framed by Narmour primarily as perceptual-cognitive phenomena, the I-R patterns discussed in Section 6 have an innate physicality. They motivate an image-schematic translation and an embodied response, such that—to take the several instances of **P** in M*a*–M*d* as an example [Figure 5, (ii)]—a falling line conjures up the impression of an object in descent; and it motivates the head and body to tilt downwards, as if to trace and follow the object's path. Similarly, a **(VR)**, such as occurs in M*g* [Figure 5, (iii)], gives the impression of the final stage of an ascent to a peak, followed by a precipitous fall; and it similarly motivates an upward then downward shift of the body's center of gravity. Thus, the I-R patterns, and the musemes that encompass them, motivate indirect physical “tracking” actions—mirrors, or echoes, of the music, translated to bodily motion—and these actions help us to understand the music physically, as opposed to cognitively, insofar as these intimately connected domains can be distinguished. That these are largely innate responses helps explain the group synchrony discussed in Section 3—if one person responds in this way to a musical line, others are likely to respond similarly, the behavior being reinforced by positive feedback fostered by cultural transmission. Perceptual-cognitive … embodied linkages also engage an additional component, especially with singing-style musemes: a degree of pseudo-vocalisation on the part of the receiver (and sometimes the producer^28^) in which the vocal tract silently traces the contour of the melodic line by tensing and relaxing the vocal cords in such a way as to “prime” the required pitches, but often without the expiration of air that would produce audible vocalisations (Bruder and Wöllner, 2021).

Making a distinction between producers (performers) and receivers (listeners), some embodied behaviors are overt, being directly associated with the music's production, such as the coordination of the lungs, vocal cords, and tongue in singing, or the movement of the fingers on keyboards. In this sense, they are correlated with the “digital” (primary-parameter; rhythm-pitch) aspects of the music—producing the right notes in the correct temporal order. Other embodied behaviors are indirectly related to the music's production, serving to emphasize and add nuance to the framework of pitch and rhythm (Snyder, 2000, p. 86). In this sense, they are correlated with the “analog” (secondary-parameter; tempo-timbre-dynamic) aspects of the music.^29^ Beyond the mechanics of musical production, much embodied motion, certainly on the part of the receiver, is often very slight (covert) and thus generally imperceptible. Yet it represents a clear example of the power of musemes to motivate physical movement, in that (as noted in Section 3), a museme does not just code for artifacts—such as the physical sound of the music, in the form of sound-waves moving through the air, or as printed scores—but also for behaviors that give rise to (production), and that result from (reception), those artifacts (see also the discussion of the interconnection of gestemes and choreoemes with musemes in Section 2).

As suggested above, our brain-based perception and cognition of a museme is connected to, indeed inseparable from, our image-schematic and embodied cognition of it as a pattern for singing or dancing. Moreover, there appears to exist a museme-embodiment constellation [to borrow the terminology of Merker (2012)] that connects—to list in reverse—the vocal/physical motions (v) resulting from the embodied responses (iv) to the image-schematic translation (iii) of the I-R structures (ii) of musemes (i).^30^ As is hypothesized in Section 3, musemes that accord with the singing or brilliant styles interact with the other elements of the constellation in different ways. Insofar as they can be separated, the differences between musemes articulating the two styles, and their panoply of evolutionary, psychological, and cultural associations, is summarized in Table 1.

**Table 1:** Summary of differences between singing-style and brilliant-style musemes.

**Singing style**	**Brilliant style**
Mainly conjunct	Often disjunct
Narrow tessitura	Wide tessitura
Even rhythm	Uneven rhythm
Measured pace	Faster pace
Melodic	Rhythmic
Singing	Dancing
Prosodic	Tactus-driven/metrical
Characterized predominantly by **P** and associated I-R structures	Characterized predominantly by **R** and associated I-R structures
Linear/teleological	Circular/cumulative
Affective/emotional	Energizing/motivating
Evolutionarily younger?	Evolutionarily older?
Primarily cortical	Primarily cerebellar
Associated with small hominin group interactions (mother-infant bonding, mate-attraction)?	Associated with larger/communal hominin group interactions (group bonding)?

Of course, Table 1 encapsulates multiple open hypotheses that stretch far back into the evolutionary history of our species. The short musical analysis offered here is insufficient in itself to evidence even a small subset of them—and to elucidate their relationship to the rest of the aforementioned constellation—not least because it has only identified a small sample of musemes in just two works. Moreover, as two vocal works, it is unsurprising that the overt incursion of brilliant-style musemes is relatively limited in MC and TWTIA. Clearly, further research is needed, involving a larger sample of musical repertoire, and empirical studies of listener responses, possibly supported by neuroimaging studies and studies involving the correlations between musemes and lexemes.

Nevertheless, as a small step in this direction, certain aspects of the constellation can be observed in another potential source of evidence—the videos of TWTIA (Appendix 2, Table 3) and of MC referred to in Section 4.1 and Section 5, respectively. In the TWTIA video, and while there are certainly exceptions, there is also a clear, if subtle, shadowing effect, whereby tiny bodily movements trace the contours of M*f-a* + M*a*–M*f-c* + M*c*, M*d* and M*g*, feeling not only their musical ascent and descent in terms of I-R structures and their resultant image schemata and embodied effects—rising up and then sinking down into acceptance of the outcome of the game—but also the “pain-pleasure” effect of the appoggiatura-resolution/pain-pleasure sequences that constitute M*a*–M*d*.^31^ A similar dynamic is at play in MC, where several interpretations—such as that of Elīna Garanča (Saint-Saëns, 2011) referred to in relation to Figure 3—correlate the contours of the musemes constituting the MC-TWTIA Musemeplex (and, indeed, of other musemes not considered here, such as those in Phrases 1 and 2 of Section B), and explicit, if subtle, rising and falling gestures.

A final issue here relates to the nature-nurture dichotomy—to the relationship between the innate behaviors driving IR → image-schematic → embodied linkages and the culturally transmitted nature of musemes. There are in the final part of the TWTIA video, at 4:11–4:55 (Outro), soft-focus close-ups on Lyngstad, then Andersson, then Ulvaeus, then Lyngstad. These are synchronized with statements of M*a*, M*b*, M*c*, and M*d*, respectively. In conformity with the point above, and in contrast to the haziness of their soft-focus halo, each of the three singers enunciates the words clearly, reinforcing the attacks on the four pitches of these musemes by various subtle body and head movements that fractally reiterate the overall downward motion of the four musemes. This is perhaps most strongly the case with Andersson and Ulvaeus, who emphasize the word “loser” with particularly clear head movements. Broadly, the singers' actions constitute an overt assent gesture—yes, the winner really *does* take it all—and the nod-like head movements appear to be versions of the near-universal (innate?) “yes” downward head-gesture that is associated image-schematically with these four musemes. This segment illustrates the complex imbrication here of the natural/innate (genetic) and the nurtural/learned (memetic). For the former, and in addition to the musemes' intrinsic image schemata and embodied phemotypes, the action of enunciating the word implies a slight downward inclination of the head, which serves as a token of the word in silent gestures–nods–of agreement. For the latter, parents permit, or indeed prohibit, certain behaviors in their children with exaggerated head-nods and head-shakes that children appear readily to replicate in similarly mannered ways.

Thus, such gestures appear to be socially transmissible; that is, they are gestemes. Moreover, and further to the discussion in Section 2, the I-R structure of a museme might motivate certain image-schematic forces that, when embodied, create certain gestures. These gestures may then go on to be replicated independently of the natural forces that initially gave rise to them. But it is important to note that when a museme is associated with a verbal-conceptual meme articulating a positive (yes; head down) or negative (no; head left-to-right) sentiment, then it is likely that natural/innate—not nurtural/learned—constraints will tend to motivate head-nodding or head-shaking, respectively. Nevertheless, such gestures appear not to be wholly innate—there is a memetic component—and in support of this one might cite examples where such texts are *not* associated with the expected gesture. A case in point is a performance of TWTIA by Edsilia Rombley on the Dutch television programme *De Beste Zangers van Nederland* (ABBA, 2014), where, at 2:25–2:29 and 2:32–2:35, the backing singers in the Outro (analogous to the roles of Lyngstad, Andersson, and Ulvaeus in ABBA 1980b) do not (as far as the brevity of the cut-aways to them permits one to determine) replicate gestures similar to those of the three ABBA members in the passage discussed above. Because these three members of ABBA appear to have acquired aspects of these gestures from each other, through the process or producing and rehearsing the song and video of TWTIA; and because Rombley's backing singers do not utilize similar gestures, despite presumably knowing the TWTIA video, it is likely that the innate component of museme-associated gestures does not wholly regulate their learned dimension. To paraphrase and contradict Wilson's famous assertion, genes do *not* (invariably) hold culture on a leash (Wilson, 1978, p. 167).

## 8 Conclusion: memetic drive as shaper of vocalisation and rhythmicisation

I have sketched, in brief, the outlines of a model of musical pattern replication made up of a constellation of interconnected aspects. Drawing on the theory of memetics (Section 2), I have made a distinction between musemes in the singing style and those in the brilliant style (Section 3), arguing that each category implicates distinct (but partially overlapping) evolutionarily shaped pathway for its perception and cognition. Using a particularly salient case of museme replication linking music from two cultural worlds and different centuries (Section 4 and Section 5), I have argued that the perceptual-cognitive salience of musemes, their fecundity, is partly conditional on their Implication-Realisation structure (Section 6). Finally, I have argued that the I-R structure, as a representation of the museme's motion-vector, activates an image-schematic perception that triggers the embodied cognition of the museme, which then—going full-circle—motivates a pseudo-vocalisation and/or a gesturalisation of the museme that reconnects it to its original evolutionary motivations (Section 7). It will be understood that my focus on museme-replication here has been decidedly “microcosmic,” looking only at a small collection of musemes hypothesized to have been replicated across just two works. For a more “macrocosmic” methodology, Anglada-Tort et al. (2023) offers a corpus-based approach, exploring nature-nurture interactions in song transmission. This, and cognate rhythm-simulation experiments such as Ravignani et al. (2017), parallel those conducted in the realm of language (Scott-Phillips and Kirby, 2010; Kirby, 2013), and they suggest the importance of reconciling big-data and computer-simulation approaches to understanding the biological and cultural evolution of musicality and music with more musicological and music-theoretical/analytical approaches based on fine-grained tracing of pattern relationships across specific works, such as in the present study.

The previous section has indicated that there is a complex process of gene-meme coadaptation occurring in music, whereby musemes (via I-R and image-schematic factors) motivate embodied behaviors that, while essentially natural/innate, give rise to gestures that escape this constraint and go on to be culturally transmitted, as nurtural/learned phenomena—as gestemes. These complex associations of musemes, verbal-conceptual memes, and gestemes “seek” (in the metaphorically intentionalist language of meme theory) to optimize their replicative chances in ways that *ceteris paribus*—and in conjunction with the other (natural/innate) elements of the constellation discussed in Section 7—are “intended” to enhance their selective advantage in the wider meme pool over time. As a final issue, and to offer another hypothesis that this article does not have sufficient space to consider, let alone evidence, in full, I consider the possibility that memes have the potential to shape the genetic environment in which they exist. That is, and to pick up Wilson's metaphor from the end of Section 7, memes may have the capacity either to stretch the leash, to break it, or even to reverse it, giving themselves mastery over genes.

Such a possibility has been theorized in Blackmore's (1999, p. 76–80; 2000, p. 31–33; 2001, p. 243–245) “memetic drive” model, whereby memes push genes toward the increased encephalisation necessary for their imitation and storage. I give a full overview in Jan (2022, p. 253–258) but Blackmore's process can be summarized, after that overview, as follows. Note that references to memes below potentially encompass all the categories of cultural replicator outlined in the final paragraph of Section 2.

1. *Selection for Imitation*: “Capacity-to-imitate” (CtI) genes (controlling the perceptual-cognitive and vocal-motor substrates for imitation) will tend to spread in a gene-pool because of the fitness advantages imitation confers on an individual compared with trial-and-error learning (Blackmore, 1999, p. 77). Those most adept at imitation are termed “meme fountains” (“MF”) by Blackmore (2000, p. 32). This mechanism alone can explain an increase in brain size, because it binds encephalisation to survival advantage via Darwinian natural selection (Blackmore, 2000, p. 32). This is because imitation requires substantial brain capacity; those with the biggest brains will tend to be the best imitators and will tend, via the survival advantage imitation-transmitted knowledge confers, to have more viable offspring.^32^
2. *Selection for Imitating the Imitators*: A genetically controlled ability to identify and preferentially imitate MFs may confer a “borrowed” gene-fitness advantage on this ability-detector's possessor, leading to a differential increase of such “imitate-the-meme-fountains” genes (Blackmore, 1999, p. 77–78). Memetic evolution and the expansion of culture gathers pace in this phase (Blackmore, 2000, p. 32), perhaps engendering, among other replicator-types, the early musemes (“protemes”) of musilanguage.
3. *Selection for Mating with the Imitators*: Here, advantages to genes and advantages to memes diverge. While the imitation described in the stages above would probably have been built on a substrate of innate capacities that arose initially via natural selection to fulfill a number of functions, it may subsequently have been augmented by sexual selection (Dennett, 2017, p. 266), leading to the appearance of coevolutionary sexual selection. As with all coevolutionary processes a replicator's interests are sometimes best served not by continued cooperation but by competition:
  - From the point of view of genes: (i) it is advantageous for a female to mate with a male MF because of the fitness advantages (accruing from a high capacity to imitate memes) conferred on her offspring by the CtI genes (Blackmore, 1999, p. 78–79). As an instance of sexual selection, this preferential mating process will tend to lead not only to a differential increase of CtI genes (the “ornament”), but also of “mate-with-the-meme-fountains” genes (the “preference”). Moreover, (ii) there will be an *enhanced* advantage for any alleles of the CtI genes that privilege replication of the most currently “favored” memes (Blackmore, 1999, p. 80)—assuming such memes are initially gene-replication-enhancing—and, thus, an associated advantage for females to mate with those males with these specific alleles.
  - From the point of view of memes, this initially gene-beneficial privileging of the most “favored” memes will initiate a process whereby: (i) memetic evolution is further expedited, in the form of ever more diverse and extreme ornaments; (ii) the ornament-memome may give rise to an ornament-phemotype that is detrimental to the replication of genes; and (iii) such gene-detrimental ornaments will tend to evolve much more rapidly than genes can evolve to control them, meaning that memes, capitalizing on genetically mediated preferences, are able to “outwit” genes (Blackmore, 1999, p. 78). In this sense, memetic evolution has escaped the genes' leash and is harnessing increased encephalisation to its own ends (Blackmore, 1999, p. 80).

If valid [and some studies have supported it (Jan, 2022, p. 257–258)], it is not difficult to see how the specific types of imitation considered in this article—those facilitating the vocalisations underpinning the singing style and the rhythmicisations underpinning the brilliant style—could have been supercharged by memetic drive. The account offered here therefore opens up insights only a dual-replicator perspective can afford: music is seen as changing (but also to some extent staying the same, as MC and TWTIA indicate) through X-eme-driven cultural evolution in a way that is constrained by the gene-shaped capacities—for singing and for dancing—underpinning musicality. Yet those very X-emes appear to have shaped these capacities in ways that, while benefiting genes through the social-bonding adaptive benefits conferred by musicality, nevertheless allow them to slip those bonds and pursue quasi-independent replicative trajectories.

## Data availability statement

The original contributions presented in the study are included in the article/Supplementary material, further inquiries can be directed to the corresponding author.

## Author contributions

SJ: Writing – original draft.

## Footnotes

^1^I alighted upon the term museme independently of Tagg (2016), conflating “music” and “meme” in an example of convergent evolution (*homoplasy*, i.e., alighting upon the same design solution independently), as opposed to direct transmission (*homology*, i.e., similarity owing to evolutionary connection) (Ridley, 2004, p. 427–428, 480). While there are alignments between our uses of the term, mine is distinguished from Tagg's by its specifically evolutionary, as opposed to his semiotic, focus—as a unit of cultural selection in music.

^2^I use the term “work” very cautiously here, aware of the considerable problems of applying the “work concept” (Goehr, 1992) to popular music, with its commonly multi-author, collaborative mode of genesis; and its generally descriptive (“the performers did this”) rather than prescriptive (“the performers should do this”) conventions of notation.

^3^The practice of circular breathing, found in such ancient traditions as Australian didjeridu playing, nevertheless affords a means of transcending the constraints of lung capacity in external aerophones.

^4^The earliest known flutes sounded one or two octaves higher than the female and male human voices, respectively, and only later, as manufacturing technologies became more sophisticated, approached the natural ranges of human voices.

^5^A step is here defined as the interval of a (major or minor) second. A skip is defined as any interval larger than a second. In terms of the harmonic series, skips precede steps, and the low-ratio skips—octaves, fifths and fourths—are fundamental to many musics. See also Bannan et al. (2024).

^6^The Italian designation *cantabile* (designating the attribute of being “singable” in instrumental music) appears as early as Zarlino's treatise *Le istitutioni harmoniche* of 1,558 (Fallows, 2001).

^7^Hearing the playing of the virtuoso Anton Stadler (1753–1812), Mozart observed the affinity between the clarinet and the human voice (Hacker, 1969, p. 359). See also footnote **21**.

^8^This mirrors the accounts given by theorists of the thematic structure of sonata-form movements—a structural model that became the foundational organisational principle of much European music in the eighteenth century—in that the first subject is often described as rhythmically energetic and based on arpeggio patterns (therefore disjunct); whereas the second subject is often described as lyrical (therefore conjunct). That the former is also often described as “masculine” and the second as “feminine” might not wholly be due to the sexism of (predominantly male) music theorists (Webster, 2001).

^9^These elements are themselves of memetic significance in terms of indicating the style and distinct character of ABBA's performance. Indeed, this illustrates the hierarchical nature of memetic processes in music, with several distinct elements being dependent on each other at different levels of our psycho-acoustic processing. Nevertheless, to explore this is beyond the scope of this article.

^10^A fuller analysis of TWTIA, expanding upon the points given below and drawing in music other that the ABBA song and the Saint-Saëns duet, is available at https://stevenbjan.com/. The lyrics of TWTIA, an overview of the video, and the libretto of MC, are given in Appendixes 1–3, respectively.

^11^A secondary dominant is a chord that acts as a local, temporary dominant in relation to a degree of the scale other than the tonic (I), whose (primary) dominant is V. Thus, the (secondary) dominant of chord ii in a major key is chord VI, provided the third of the latter chord is raised. In a common convention, I am using upper-case Roman numerals here to designate major chords and lower-case Roman numerals to designate minor chords.

^12^While primarily related to the issues covered in Section 6, Figure 5 also serves, secondarily, as a summary list of all the musemes discussed in this article. While M*e* and M*h* are not directly relevant to Figure 5, they are included, as Figure 5 (v), for this secondary, summary, purpose.

^13^In evolutionary-psychological terms, the appoggiatura, or “leaning note”, represents a tendency toward resolution, whereby a note foreign to the prevailing harmony, and thus unstable/open, moves up or down to find repose on a stable/closed harmony note. While the tendency to favor closure has a gestalt (and thus natural) component, there is also a nurtural dimension regulating the treatment of this and other “inessential” notes, one that incorporates learned aspects of harmonic syntax. Other instances of ABBA's use of the (often 4–3) appoggiatura include the final cadence of the verse of “Slipping Though my Fingers” (at “funny little girl”); and the final cadence of the chorus of “Our Last Summer” (at “mem'ries that remain”), to list just two.

^14^In this article, “b.” indicates bar (or measure) number. Superscript numerals after a bar number are used to indicate the beat, or beat-range, upon which an event occurs. Thus, “b. 1^2^” designates the second beat (of four) of the first bar, and “b. 2^3–4^” designates the third and fourth beats, inclusive, of the second bar.

^15^By convention, the seven degrees (steps) of the major or minor scale are indicated using numbers with the caret symbol ($\hat{)}$ placed above them. For example, $\hat{5}$ in the key of G♭ major represents the note D♭, the fifth degree of the scale of G♭ major.

^16^Not to be confused with the superscripts used to designate the beat-position of an event in a bar, a superscript after a note's letter name is used to indicate the octave in which that note occurs, using the standard “Helmholtz” system. Here, “middle” C = c^1^, and the C an octave above this = c^2^. The F♯ half way between these two notes is therefore f♯^1^, etc. Note names given in capital letters refer here to non-register-specific pitches.

^17^The galant style dominated European music during the period *c*. 1720–*c*. 1780 (Heartz, 2003), and was characterized by its rich lexicon of standardized (schematic) patterning (Gjerdingen, 1988, 2007; Byros, 2009; Jan, 2013; Rawbone and Jan, 2020).

^18^As with M*a*, M*b*, M*c*, and M*d*, this pattern occurs four times in the verse, in its bb. 1–2, bb. 3–4, bb. 5–6, and bb. 7–8. The second, third, and fourth iterations are not given individual “M” labels here (unlike M*a*, M*b*, M*c*, and M*d*), owing to the focus on the Intro/Chorus/Outro in discussing the similarities with the Saint-Saëns duet; but a fully comprehensive labeling system would assign them separate appellations, owing to their individual pitch, scale-degree, and intervallic structures.

^19^One might, however, argue that some genres of opera functioned, in their own time, as popular music; and that the extensive consumption of opera on platforms such as *YouTube* makes it popular music today.

^20^Nevertheless, there are no references to opera, Saint-Saëns, *Samson et Dalila*, or MC in Palm (2014), which suggests that, while Andersson and Ulvaeus had facility in music notation (unlike many popular musicians), they had no great familiarity with the European classical tradition beyond perhaps knowing such “popular classics” as MC. I have not as yet been able to contact Andersson or Ulvaeus to ask them whether the connections hypothesized in this article were ones they were consciously aware of at the time of the composition of TWTIA. See also footnote **22**.

^21^It is interesting that the clearest statement of the Phrase-3 theme emerges in the wistful clarinet solos—see footnote **7**—linking B^1^ and A^2^ (bb. 46–49) and closing B^2^ (bb. 97–100), during which Samson declaims “Dalila! Dalila! Je t'aime!”. I am grateful to Nicholas Bannan for this point.

^22^Further to footnote **20**, it is not necessarily the case that the pattern replication hypothesized here was undertaken intentionally/consciously by the composers involved. Memetics simply hypothesizes that perceptually and cognitively salient patterns will tend to be copied from brain to brain, whether the owners of those brains are aware of what is going on or not. Determining whether the similarities between MC and TWTIA are the result of conscious or unconscious processes is not the point: the memetic similarities are there to be observed. Nevertheless, it seems likely that (replication-transmitted) homologies have a greater chance of being consciously motivated than (good-trick) homoplasies (see footnote **1**).

^23^Inspired by aspects of Gestalt psychology, much of Narmour's (1990, p. 35–40) work considers the innate perceptual-cognitive constraints that shape the unfolding of musical material within a work (the intraopus perspective, after Meyer) and, by implication, its cultural evolution over time (the extraopus perspective, analogous to Meyer's dialect). His broadly inductive/empirical approach contrasts with the deductive/axiomatic approach of Schenker (Narmour, 1977): for Schenker, the “background-level” *Ursatz* shapes the unfolding of material at lower structural-hierarchic levels; for Narmour, higher-level structure emerges from lower-level implications and realisations.

^24^While beyond what is necessary for present purposes, Narmour (1990, p. 426) has strict definitions of what constitute small and large intervals in specific circumstances.

^25^Here, and in **VR**, “**V**” stands for “vector”, referring to the mathematical concept of a geometrical object possessing magnitude and direction (Narmour, 1990, p. 90).

^26^Retrospective structures [symbolized by Narmour with “( )”] involve, as their name implies, an element of *post-hoc* (re-)cognition of what has just been heard (Narmour, 1990, p. 203–206).

^27^Schenker's is an *a priori*/top-down/deductive theory, in that the “fundamental structure,” situated at the background level, is hypothesized to regulate the unfolding of patterns at lower structural levels. Narmour's, by contrast, is an *a posteriori*/bottom-up/inductive theory, in that the realisation of lower-level implications is held to be the foundation upon which higher-level implications are built. A pragmatic compromise between these extremes holds that both axiomatic and empirical forces regulate the production and reception of music and that both categories are complex interactions between natural and nurtural factors (Jan, 2010, p. 13).

^28^Examples of this include the overt vocalisations by Glen Gould during piano performances.

^29^A distinction from Meyer(1996, p. 14–16), pitch and rhythm are assigned the status of primary parameters because they are discrete and measurable—a given C♯ might be played too sharp or too flat in a particular performance, but categorical perception (Calvin, 1998, p. 68) means that it is assigned by a listener to a single, digital, pitch- or note-class. Timbre and dynamics, by contrast, are assigned the status of secondary parameters by Meyer because they are difficult to discretise—the difference between *f* (*forte*) and *ff* (*fortissimo*) is relative, not absolute. In Meyer's view, the former class of parameters is more capable of supporting syntax in music than the latter.

^30^Note that this constellation traces a continuum from the necessarily digital, logocentric taxonomy of musemes to their analog translations into embodied effects.

^31^The opposite of such subtle, micro-gestures is represented, if the historical accounts are to be believed, by the eighteenth-century singer Adriana Ferrarese del Bene (*c*. 1755–*c*. 1804), the first Fiordiligi in Mozart's opera *Così fan tutte* K. 590 (1790). Her apparent tendency to throw her head backwards on rising intervals and to throw it forward on falling ones has been suggested as the motivation of her mocking aria “Come scoglio” in that opera (Mann, 1986, p. 542).

^32^This stage is “a version of the Baldwin effect …, which applies to any kind of learning—once some individuals become able to learn something, those who cannot are disadvantaged and genes for the ability to learn, therefore, spread” (Blackmore 2001, 243–244; see also Podlipniak 2017).

## References

[B1] ABBA (1980a). Super Trouper. New York, NY: Epic.

[B2] ABBA (1980b). “The winner takes it all” music video. Available online at: https://www.youtube.com/watch?v=92cwKCU8Z5c

[B3] ABBA (2014). “The winner takes it all”: Edsilia Rombley. Available online at: https://www.youtube.com/watch?v=7mVTv8o4qHA

[B4] Andersson B. Ulvaeus B. (1999). Mamma Mia! Vocal Selections. Sulphur, LA: Wise Publications.

[B5] Andersson B. Ulvaeus B. Johnson C. (1999). Mamma Mia! The Musical.

[B6] Anglada-Tort M. Harrison P. M. C. Lee H. Jacoby N. (2023). Large-scale iterated singing experiments reveal oral transmission mechanisms underlying music evolution. Curr. Biol. 33, 1472–1486. 10.1016/j.cub.2023.02.070 36958332

[B7] Azumagakito T. Suzuki R. Arita T. (2018). An integrated model of gene-culture coevolution of language mediated by phenotypic plasticity. Scient. Rep. 8, 1–11. 10.1038/s41598-018-26233-7 29795297 PMC5966417

[B8] Bannan N. Bamford J. S. Dunbar R. I. M. (2024). The evolution of gender dimorphism in the human voice: the role of octave equivalence. Curr. Anthropol. 10.31234/osf.io/f4j6b

[B9] Bigand E. (1993). Contributions of music to research on human auditory cognition, in Thinking in Sound: The Cognitive Psychology of Human Audition, eds. S. McAdams and E. Bigand (Oxford: Clarendon Press), 231–277. 10.1093/acprof:oso/9780198522577.003.0008

[B10] Blackmore S. J. (1999). The Meme Machine. Oxford: Oxford University Press.

[B11] Blackmore S. J. (2000). The memes' eye view, in Darwinizing Culture: The Status of Memetics as a Science, ed. R. Aunger (Oxford: Oxford University Press), 25–42. 10.1093/acprof:oso/9780192632449.003.0002

[B12] Blackmore S. J. (2001). Evolution and memes: the human brain as a selective imitation device. Cybern. Syst. 32, 225–255. 10.1080/019697201300001867

[B13] Brooker G. Reid K. Fisher M. (1967). A Whiter Shade of Pale. London: Deram Records.

[B14] Brown S. (2000). The ‘musilanguage' model of musical evolution, in The Origins of Music, eds. N. L. Wallin, B. Merker, and S. Brown (Cambridge, MA: MIT Press), 271–300. 10.7551/mitpress/5190.003.0022

[B15] Brown S. (2022). Group dancing as the evolutionary origin of rhythmic entrainment in humans. New Ideas Psychol. 64, 1–12. 10.1016/j.newideapsych.2021.100902

[B16] Bruder C. Wöllner C. (2021). Subvocalization in singers: laryngoscopy and surface EMG effects when imagining and listening to song and text. Psychol. Music 49, 567–580. 10.1177/0305735619883681

[B17] Byros V. (2009). Towards an “archaeology” of hearing: schemata and eighteenth-century consciousness. Musica Humana 1, 235–306.

[B18] Calvin W. H. (1998). The Cerebral Code: Thinking a Thought in the Mosaics of the Mind. Cambridge, MA: MIT Press. 10.7551/mitpress/1775.001.0001

[B19] Carney J. (2020). Thinking avant la lettre: a review of 4E cognition. Evolut. Stud. Imagin. Cult. 4, 77–90. 10.26613/esic.4.1.172 32457930 PMC7250653

[B20] Conard N. J. Malina M. Münzel S. C. (2009). New flutes document the earliest musical tradition in southwestern Germany. Nature 460, 737–740. 10.1038/nature08169 19553935

[B21] Cook N. Clarke E. Leech-Wilkinson D. Rink J. (2009). The Cambridge Companion to Recorded Music. Cambridge: Cambridge University Press. 10.1017/CCOL9780521865821

[B22] Cope D. (2003). Computer analysis of musical allusions. Comput. Music J. 27, 11–28. 10.1162/01489260360613317

[B23] Cox A. (2016). Music and Embodied Cognition: Listening, Moving, Feeling, and Thinking. Bloomington, IN: Indiana University Press. 10.2307/j.ctt200610s

[B24] Dawkins R. (1983). Universal darwinism, in Evolution From Molecules to Men, ed. D. S. Bendall (Cambridge: Cambridge University Press), 403–425.

[B25] Dawkins R. (1989). The Selfish Gene. Oxford: Oxford University Press, 2 edition.

[B26] Dennett D. C. (1995). Darwin's Dangerous idea: Evolution and the Meanings of Life. London: Penguin.

[B27] Dennett D. C. (2017). From Bacteria to Bach and Back: The Evolution of Minds. London: Penguin.

[B28] Dissanayake E. (2012). Art and Intimacy: How the Arts Began. Seattle, WA: University of Washington Press.

[B29] Fallows D. (2001). Cantabile, in Grove Music Online, ed. D. L. Root. Available online at: http://www.oxfordmusiconline.com/subscriber/article/grove/music/04746

[B30] Fux J. J. (1965). The Study of Counterpoint. From Johann Joseph Fux's Gradus ad Parnassum. New York: W. W. Norton & Company.

[B31] Gjerdingen R. O. (1988). A Classic Turn of Phrase: Music and the Psychology of Convention. Philadelphia, PA: University of Pennsylvania Press.

[B32] Gjerdingen R. O. (2007). Music in the Galant Style. Oxford: Oxford University Press. 10.1093/oso/9780195313710.001.0001

[B33] Goehr L. (1992). The Imaginary Museum of Musical Works: An Essay in the Philosophy of Music. Blackstone, VA: Clarendon Press.

[B34] Hacker A. (1969). Mozart and the basset clarinet. Musical Times 110, 359–362. 10.2307/951470

[B35] Heartz D. (2003). Music in European Capitals: The Galant Style 1720–1780. New York: Norton.

[B36] Hornbostel E. M. v. Sachs C. (1914). Systematik der Musikinstrumente: Ein Versuch. Zeitschrift für Ethnol. 46, 553–590.

[B37] Huron D. (2001). Tone and voice: a derivation of the rules of voice-leading from perceptual principles. Music Percept. 19, 1–64. 10.1525/mp.2001.19.1.1

[B38] Jan S. B. (2003). The evolution of a “memeplex” in late Mozart: Replicated structures in Pamina's “Ach ich fühl's”. J. R. Musical Assoc. 128, 329–370. 10.1093/jrma/fkg002

[B39] Jan S. B. (2007). The Memetics of Music: A Neo-Darwinian View of Musical Structure and Culture. Farnham: Ashgate.

[B40] Jan S. B. (2010). Memesatz contra Ursatz: Memetic perspectives on the aetiology and evolution of musical structure. Musicae Scient. 14, 3–50. 10.1177/102986491001400101

[B41] Jan S. B. (2013). Using galant schemata as evidence for universal Darwinism. Interdisc. Sci. Rev. 38, 149–168. 10.1179/0308018813Z.00000000042

[B42] Jan S. B. (2014). Similarity continua and criteria in memetic theory and analysis. J. Music Res. 5, 125. Available online at: http://www.jmro.org.au/index.php/mca2/article/view/125

[B43] Jan S. B. (2016). A memetic analysis of a phrase by Beethoven: Calvinian perspectives on similarity and lexicon-abstraction. Psychol. Music 44, 443–465. 10.1177/0305735615576065

[B44] Jan S. B. (2022). Music in Evolution and Evolution in Music. Cambridge: Open Book Publishers. 10.11647/OBP.0301

[B45] Jeppesen K. (1992). Counterpoint: The Polyphonic Vocal Style of the Sixteenth Century. Mineola: Dover.

[B46] Kirby S. (2013). Transitions: The evolution of linguistic replicators, in The Language Phenomenon: Human Communication from Milliseconds to Millennia, eds. P. M. Binder, and K. Smith (Cham: Springer), 121–138. 10.1007/978-3-642-36086-2_6

[B47] Klein M. L. (2005). Intertextuality in Western Art Music. Bloomington, IN: Indiana University Press.

[B48] Koch W. A. (1986). Evolutionary Cultural Semiotics. Washington, DC: N. Brockmeyer.

[B49] Korsyn K. (1991). Towards a new poetics of musical influence. Music Anal. 10, 3–72. 10.2307/853998

[B50] Kunej D. Turk I. (2000). New perspectives on the beginnings of music: Archeological and musicological analysis of a middle paleolithic bone ‘flute', in The Origins of Music, eds. N. L. Wallin, B. Merker, and S. Brown (London: MIT Press), 235–268. 10.7551/mitpress/5190.003.0020

[B51] Leman M. (2008). Embodied Music Cognition and Mediation Technology. London: MIT Press. 10.7551/mitpress/7476.001.0001

[B52] Lloyd P. (2008). Mamma Mia! The Movie.

[B53] Lynch A. (1998). Units, events and dynamics in memetic evolution. J. Memet. 2, 5–39.

[B54] Mann W. (1986). The Operas of Mozart. London: Cassell.

[B55] Mattheson J. Harriss E. C. (1981). Johann Mattheson's Der vollkommene Capellmeister: A Revised Translation With Critical Commentary. Studies in Musicology Volume 21. Ann Arbor: UMI Research Press.

[B56] Matzinger T. Fitch W. T. (2021). Voice modulatory cues to structure across languages and species. Philos. Trans. R. Soc. B 376, 1–11. 10.1098/rstb.2020.0393 34719253 PMC8558770

[B57] McClary S. (2004). Rap, minimalism, and structures of time in late twentieth-century culture, in Audio Culture: Readings in Modern Music, eds. C. Cox, and D. Warner (London: Continuum), 289–298.

[B58] Merchant H. Grahn J. Trainor L. J. Rohrmeier M. Fitch W. T. (2018). Finding the beat: A neural perspective across humans and nonhuman primates, in The Origins of Musicality, ed. H. Honing (Cambridge, MA: MIT Press), 171–203. 10.7551/mitpress/10636.003.0013

[B59] Meredith D. (2016). Computational Music Analysis. New York: Springer. 10.1007/978-3-319-25931-4

[B60] Merker B. (2000). Synchronous chorusing and human origins, in The Origins of Music, eds. N. L. Wallin, B. Merker, and S. Brown (Cambridge, MA: MIT Press), 315–327. 10.7551/mitpress/5190.003.0024

[B61] Merker B. (2012). The vocal learning constellation: Imitation, ritual culture, encephalization, in Music, Language, and Human Evolution, ed. N. Bannan (Oxford: Oxford University Press), 215–260. 10.1093/acprof:osobl/9780199227341.003.0009

[B62] Meyer L. B. (1973). Explaining Music: Essays and Explorations. Chicago: University of Chicago Press. 10.1525/9780520333109

[B63] Meyer L. B. (1996). Style and Music: Theory, History, and Ideology. Chicago: University of Chicago Press.

[B64] Miller G. (2000). Evolution of human music through sexual selection, in The Origins of Music, eds. N. L. Wallin, B. Merker, and S. Brown (Cambridge, MA: MIT Press), 329–360. 10.7551/mitpress/5190.003.0025

[B65] Miller G. A. (1956). The magical number seven, plus or minus two: some limits on our capacity for processing information. Psychol. Rev. 63, 81–97. 10.1037/h0043158 13310704

[B66] Mirka D. (2014). The Oxford Handbook of Topic Theory. Oxford: Oxford University Press. 10.1093/oxfordhb/9780199841578.001.0001

[B67] Mithen S. (2006). The Singing Neanderthals: The Origins of Music, Language, Mind and Body. London: Phoenix.

[B68] Morley I. (2013). The Prehistory of Music: Human Evolution, Archaeology, and the Origins of Musicality. Oxford: Oxford University Press. 10.1093/acprof:osobl/9780199234080.001.0001

[B69] Mosing M. A. Verweij K. J. H. Madison G. Pedersen N. L. Zietsch B. P. Ullén F. (2014). Did sexual selection shape human music? Testing predictions from the sexual selection hypothesis of music evolution using a large genetically informative sample of over 10,000 twins. Evolut. Hum. Behav. 36, 359–366. 10.1016/j.evolhumbehav.2015.02.004

[B70] Narmour E. (1977). Beyond Schenkerism: The Need for Alternatives in Music Analysis. Chicago: University of Chicago Press.

[B71] Narmour E. (1990). The Analysis and Cognition of Basic Melodic Structures: The Implication-Realization Model. Chicago: University of Chicago Press.

[B72] Narmour E. (1992). The Analysis and Cognition of Melodic Complexity: The Implication-Realization Model. Chicago: University of Chicago Press.

[B73] Nattiez J.-J. (1990). Music and Discourse: Toward a Semiology of Music. Princeton: Princeton University Press.

[B74] Newen A. De Bruin L. Gallagher S. (2018). The Oxford Handbook of 4E Cognition. Oxford: Oxford University Press. 10.1093/oxfordhb/9780198735410.001.0001

[B75] Palm C. M. (2014). ABBA: Bright Lights Dark Shadows. London: Omnibus Press, 3rd edition.

[B76] Plotkin H. C. (2010). Evolutionary Worlds Without End. Oxford: Oxford University Press. 10.1093/acprof:oso/9780199544950.001.0001

[B77] Podlipniak P. (2017). The role of the Baldwin Effect in the evolution of human musicality. Front. Neurosci. 11, 542. 10.3389/fnins.2017.00542 29056895 PMC5635050

[B78] Ratner L. G. (1980). Classic Music: Expression, Form, and Style. New York, NY: G. Schirmer.

[B79] Ravignani A. (2018). Darwin, sexual selection, and the origins of music. Trends Ecol. Evol. 33, 716–719. 10.1016/j.tree.2018.07.006 30126619

[B80] Ravignani A. Delgado T. Kirby S. (2017). Musical evolution in the lab exhibits rhythmic universals. Nat. Hum. Behav. 1, 7. 10.1038/s41562-016-0007

[B81] Rawbone T. Jan S. B. (2020). The butterfly schema in the classical instrumental style: a product of the tendency for congruence. Music Anal. 39, 85–127. 10.1111/musa.12133

[B82] Ridley M. (2004). Evolution. 3 edition Hoboken: Blackwell.

[B83] Saint-Saëns C. (2011). Elīna Garanča: Camille Saint-Saëns - Aria “Mon coeur s'ouvre à ta voix”, from “Samson et Dalila”. Available online at: https://www.youtube.com/watch?v=eMadr61Wq_E

[B84] Saint-Saëns C. Lehmann J. Miller H. Wilson J. (1960). Night. Chapel Hill, NC: Brunswick Records.

[B85] Salmi R. Muñoz M. (2020). The context of chest beating and hand clapping in wild western gorillas (*Gorilla* gorilla gorilla). Primates 61, 225–235. 10.1007/s10329-019-00782-5 31894436

[B86] Savage P. E. Loui P. Tarr B. Schachner A. Glowacki L. Mithen S.. (2021). Music as a coevolved system for social bonding. Behav. Brain Sci. 44, e59. 10.1017/S0140525X20000333 32814608

[B87] Schenker H. (1979). Free composition (Der freie Satz). Harlow: Longman.

[B88] Schubert E. Pearce M. (2016). A new look at musical expectancy: the veridical versus the general in the mental organization of music, in Music, Mind, and Embodiment: 11th International Symposium, CMMR 2015, Plymouth, UK, June 16–19, 2015, eds. R. Kronland-Martinet, M. Aramaki, and S. Ystad (Berlin: Springer), 358–370. 10.1007/978-3-319-46282-0_23

[B89] Scott-Phillips T. C. Kirby S. (2010). Language evolution in the laboratory. Trends Cogn. Sci. 14, 411–417. 10.1016/j.tics.2010.06.006 20675183

[B90] Shapiro L. A. (2011). Embodied Cognition. London: Routledge. 10.4324/9780203850664

[B91] Small C. (1998). Musicking: The Meanings of Performing and Listening. Middletown, CT: Wesleyan University Press.

[B92] Snyder B. (2000). Music and Memory: An Introduction. Cambridge, MA: MIT Press.

[B93] Snyder B. (2009). Memory for music, in The Oxford Handbook of Music Psychology, eds. S. Hallam, I. Cross, and M. Thaut (Oxford: Oxford University Press), 107–117.

[B94] Spitzer M. (2004). Metaphor and Musical Thought. Chicago: University of Chicago Press. 10.7208/chicago/9780226279435.001.0001

[B95] Tagg P. (2016). Kojak: 50 Seconds of Television Music. Towards the Analysis of Affect in Popular Music. New York, NY: Mass Media Music Scholars' Press.

[B96] van der Schyff D. Schiavio A. Elliott D. J. (2022). Musical bodies, musical Minds: Enactive Cognitive Science and the Meaning of Human Musicality. Cambridge, MA: MIT Press. 10.7551/mitpress/12117.001.0001

[B97] Vanchurin V. (2020). The world as a neural network. Entropy 22, 1–20. 10.3390/e22111210 PMC7712105 33286978

[B98] Webster J. (2001). Sonata form, in Grove Music Online, ed. D. L. Root. Available online at: http://www.oxfordmusiconline.com/subscriber/article/grove/music/26197

[B99] Williams P. F. (1998). The Chromatic Fourth During Four Centuries of Music. Blackstone, VA: Clarendon Press. 10.1093/oso/9780198165637.001.0001

[B100] Wilson E. O. (1978). On Human Nature. Cambridge, MA: Harvard University Press.

