# Supplementary material for: Song and dance: a memetic angle on the evolution of musicality and music via case studies of a musemeplex in Saint-Saëns and ABBA
Source: Front Psychol. 2024 Feb 28;14:1260262. doi: 10.3389/fpsyg.2023.1260262 (PMC10935739; doi:10.3389/fpsyg.2023.1260262)
Supplement: Supplementary file 1 [file Data_Sheet_1.pdf]

APPENDIXES

Appendix 1: Lyrics of “The Winner Takes It All”

The lyrics of TWTIA concern a failed love affair, couched in terms of a game of cards, with its notions of playing by (or breaking) “the rules” of the game of love. The game is overseen, and possibly manipulated, by the gods, who play their own game of dice, in a clear reference to the trope of human actions being controlled by deities as proxies for their own desires and caprices. Table 2 shows the song’s lyrics.

Table 2. Lyrics of “The Winner Takes It All”

Verses	Choruses
**1** I don’t wanna talk<br>About things we’ve gone through<br>Though it’s hurting me<br>Now it’s history<br>I’ve played all my cards<br>And that’s what you’ve done too<br>Nothing more to say<br>No more ace to play	**1** The winner takes it all<br>The loser standing small<br>Beside the victory<br>That’s her destiny
**2** I was in your arms<br>Thinking I belonged there<br>I figured it made sense<br>Building me a fence<br>Building me a home<br>Thinking I’d be strong there<br>But I was a fool<br>Playing by the rules	**2** The gods may throw a dice<br>Their minds as cold as ice<br>And someone way down here<br>Loses someone dear<br>The winner takes it all<br>The loser has to fall<br>It’s simple and it’s plain<br>Why should I complain?
**3** But tell me does she kiss<br>Like I used to kiss you?<br>Does it feel the same<br>When she calls your name?<br>Somewhere deep inside<br>You must know I miss you<br>But what can I say?<br>Rules must be obeyed	**3** The judges will decide<br>The likes of me abide<br>Spectators of the show<br>Always staying low<br>The game is on again<br>A lover or a friend<br>A big thing or a small<br>The winner takes it all
**4** I don’t wanna talk<br>If it makes you feel sad<br>And I understand<br>You’ve come to shake my hand<br>I apologize<br>If it makes you feel bad<br>Seeing me so tense<br>No self-confidence<br>(But, you see,)	**4** The winner takes it all<br>The winner takes it all

Appendix 2: Video of "The Winner Takes It All"

The music video for TWTIA (ABBA, 1980b) is summarised in Table 3 (where "F" = (Agnetha) Fältskog, "U" = (Björn) Ulvaeus, "L" = (Anni-Frid) Lyngstad, and "A" = (Benny) Andersson). The structure of the video, in terms of the articulations between scenes/shots, broadly accords with that of the song, as indicated in the third column (the latter explained in Section 4).

Table 3. Video of "The Winner Takes It All"

Time	Action	Music
0:00–0:34	Various black-and-white stills (including images of performances) of ABBA as a quartet, plus duo-shots (including F + U, L + A, F + L), and solos, the last two being of F then U.	Intro
0:35–1:05	F singing close-up to camera, in colour, in what is revealed (at the repeat of the Verse melody) to be a café, ending with close-up to camera behind hands playing piano (F's face being reflected in the piano-lid), this overlapping with the following section.	V1
1:06–1:21	Shortly after start of this section, F walking alone by water (perhaps outside the café), followed by close-up to camera.	C1
1:22–1:51	F + U and L + A walking together happily as couples, then back to F solo looking wistfully out of the café window, then (at the repeat of the Verse melody) close-up to camera but with pensive glances away, unlike in V1.	V2
1:52–2:21	F walking by water (seen just before the start of this section), this time singing, followed by a table scene in the café where F is isolated from the conviviality of the others, then (at the repeat of the Chorus melody) close-up to camera behind hands playing piano.	C2
2:22–2:52	F solo to camera in the café, followed (at the repeat of the Verse melody) by line-up group shot in which F laughs heartily, then by group shot laughing outdoors on a grassy bank, ending briefly with the line-up group shot but with F now wistful.	V3
2:53–3:24	F close-up to camera, then (at the repeat of the Chorus melody) behind hands playing piano, and ending with F sitting isolated next to L + A and singing to camera.	C3
3:25–3:54	F close-up to camera, then walking (but not singing) by water, then (at the repeat of the Verse melody) close-up to camera behind hands playing piano, and ending briefly with the same line-up group shot of V3 but with F yet more wistful.	V4
3:55–4:10	F close-up to camera then brief outdoor shot of U with L + A walking down a street, then back to the café-table scene of C2 with U and L + A laughing and F drinking from a glass, isolated.	C4
4:11–4:55	L, then A, then U, then L in soft-focus close-ups to camera, then black-and-white stills as at the start (this time mainly ensemble shots), ending (fade-out) with F close-up to camera, silent, behind hands playing piano.	Outro

Appendix 3: Libretto of “Mon cœur s’ouvre à ta voix”

Samson et Dalila is based on the Bible’s account of the Samson and Delilah story in Judges 16, as adapted by Saint-Saëns’ librettist Ferdinand Lemaire (MacDonald, 2001). Dalila, the beautiful Philistine, uses her charms to extract the secret of the Hebrew Samson’s great strength in order that her people may defeat the Hebrews in revenge for the Philistines’ losses at the hands of the Hebrews and in the service of their god, Dagon. Articulating Samson’s conflict between duty and love – between his loyalty to the Hebrews and his growing desire for Dalila – MC is a turning point in the opera. Samson capitulates to his feelings, abandoning his duty in declaring his love for Dalila at the end of the aria’s B sections (Section 5), as Dalila’s solo becomes a duet. Thereafter, when Dalila doubts his feelings, he finally reveals his secret to her – that “[t]here hath not come a razor upon mine head; for I have been a Nazarite unto God from my mother’s womb: if I be shaven, then my strength will go from me, and I shall become weak, and be like any other man” (Judges 16:17, King James Version). Table 4 shows MC’s text with a translation (after Glaubitz, 2017).

Table 4. Text and Translation of “Mon cœur s’ouvre à ta voix”

**Section A[1]**	
Mon cœur s’ouvre à ta voix,<br>comme s’ouvrent les fleurs<br>Aux baisers de l’aurore!<br>Mais, ô mon bienaimé,<br>pour mieux sécher mes pleurs,<br>Que ta voix parle encore!<br>Dis-moi qu’à Dalila<br>tu reviens pour jamais.<br>Redis à ma tendresse<br>Les serments d’autrefois,<br>ces serments que j’aimais!	My heart opens to your voice<br>like the flowers open<br>To the kisses of the dawn!<br>But, o my beloved,<br>To dry my tears the best,<br>Let your voice speak again!<br>Tell me that to Delilah<br>You will return forever!<br>Repeat to my tenderness<br>The oaths of other times,<br>Those oaths that I loved!
**Section B[1]**	
Ah! réponds à ma tendresse!<br>Verse-moi, verse-moi l’ivresse!<br>*Samson*: Dalila! Dalila! Je t’aime!	Ah! respond to my tenderness!<br>Fill me, fill me with ecstasy!<br>*Samson*: Delilah! Delilah! I love you!
**Section A[2]**	
Ainsi qu’on voit des blés<br>les épis onduler<br>Sous la brise légère,<br>Ainsi frémit mon cœur,<br>prêt à se consoler,<br>A ta voix qui m’est chère!<br>La flèche est moins rapide<br>à porter le trépas,<br>Que ne l’est ton amante<br>à voler dans tes bras!	Like one sees the wheat<br>the blades undulate<br>Under the light breeze,<br>So trembles my heart,<br>ready to be consoled,<br>by your voice which is dear to me!<br>The arrow is less quick<br>to carry death,<br>Than is your love<br>to fly into your arms!
**Section B[2]**	
Ah! réponds à ma tendresse!<br>Verse-moi, verse-moi l’ivresse!<br>*Samson*: Par mes baisers je veux<br>sécher tes larmes,<br>Et de ton cœur<br>éloigner les alarmes,<br>Dalila! Dalila! Je t’aime!	Ah! respond to my tenderness!<br>Fill me, fill me with ecstasy!<br>*Samson*: By my kisses I want<br>to dry your tears,<br>And from your heart<br>to remove your fears,<br>Delilah! Delilah! I love you!

Glaubitz, R. (2017). Translation of “Mon coeur s'ouvre à ta voix'”. *The Aria Database*. Available online at: http://www.aria-database.com/translations/samson07_mon.txt.
Macdonald, H. (2001). “Samson et Dalila,” in *Grove Music Online*, ed. D. L. Root. Available online at: http://www.oxfordmusiconline.com/subscriber/article/grove/music/O904621.
